# Detection of cancer through exhaled breath: a systematic review

**DOI:** 10.18632/oncotarget.5938

**Published:** 2015-09-30

**Authors:** Agne Krilaviciute, Jonathan Alexander Heiss, Marcis Leja, Juozas Kupcinskas, Hossam Haick, Hermann Brenner

**Affiliations:** ^1^ Division of Clinical Epidemiology and Aging Research, German Cancer Research Center (DKFZ), Heidelberg, Germany; ^2^ Faculty of Medicine, University of Latvia, Digestive Diseases Center GASTRO, and Riga East University Hospital, Riga, Latvia; ^3^ Department of Gastroenterology, Lithuanian University of Health Sciences, Kaunas, Lithuania; ^4^ Department of Chemical Engineering and Russell Berrie Nanotechnology Institute, Technion – Israel Institute of Technology, Haifa, Israel; ^5^ Division of Preventive Oncology, German Cancer Research Center (DKFZ), Heidelberg, Germany; ^6^ German Cancer Consortium (DKTK), German Cancer Research Center (DKFZ), Heidelberg, Germany

**Keywords:** breath analysis, cancer detection, volatile organic compound, VOC, systematic review

## Abstract

**Background:**

Timely diagnosis of cancer represents a challenging task; in particular, there is a need for reliable non-invasive screening tools that could achieve high levels of adherence at virtually no risk in population-based screening. In this review, we summarize the current evidence of exhaled breath analysis for cancer detection using standard analysis techniques and electronic nose.

**Methods:**

Relevant studies were identified searching Pubmed and Web of Science databases until April 30, 2015. Information on breath test performance, such as sensitivity and specificity, was extracted together with volatile compounds that were used to discriminate cancer patients from controls. Performance of different breath analysis techniques is provided for various cancers together with information on methodological issues, such as breath sampling protocol and validation of the results.

**Results:**

Overall, 73 studies were included, where two-thirds of the studies were conducted on lung cancer. Good discrimination usually required a combination of multiple biomarkers, and area under the receiver operating characteristic curve or accuracy reached levels of 0.9 or higher in multiple studies. In 25% of the reported studies, classification models were built and validated on the same datasets. Huge variability was seen in different aspects among the studies.

**Conclusions:**

Analyses of exhaled breath yielded promising results, although standardization of breath collection, sample storage and data handling remain critical issues. In order to foster breath analysis implementation into practice, larger studies should be implemented in true screening settings, paying particular attention to standardization in breath collection, consideration of covariates, and validation in independent population samples.

## INTRODUCTION

Cancer is a leading cause of death worldwide [1]. In 2012, cancer accounted for 8.2 million deaths, and number of deaths is projected to increase to over 13 million in 2030 [2]. Early detection is essential to improve successful treatment and reduce cancer mortality and cancer screening in the asymptomatic general population might be a particularly promising approach to achieve this goal. However, only few cancer screening programs are widely used. For most deadly cancers, such as pancreatic or gastric cancer, no reliable population-based screening exists, and for other common malignancies, like breast or colorectal cancer, there is large potential for improving currently used screening methods. In particular, there is a need for reliable non-invasive screening tools that could achieve high levels of adherence at virtually no risk in population-based screening.

Breath tests might be a particularly promising approach for non-invasive cancer screening. The analysis of volatile organic compounds (VOCs) in exhaled breath can provide information on metabolic processes in the body which are modified by underlying diseases [3-5], though a detailed picture of VOCs origin is still not complete.

In this systematic review, we summarize the current evidence of exhaled breath analysis for cancer detection. Performance of different breath analysis techniques is provided for various cancers together with information on methodological issues, such as breath sampling protocol, validation of the results, and VOCs proposed as cancer-related compounds.

## RESULTS

### Literature search

**Figure 1:**
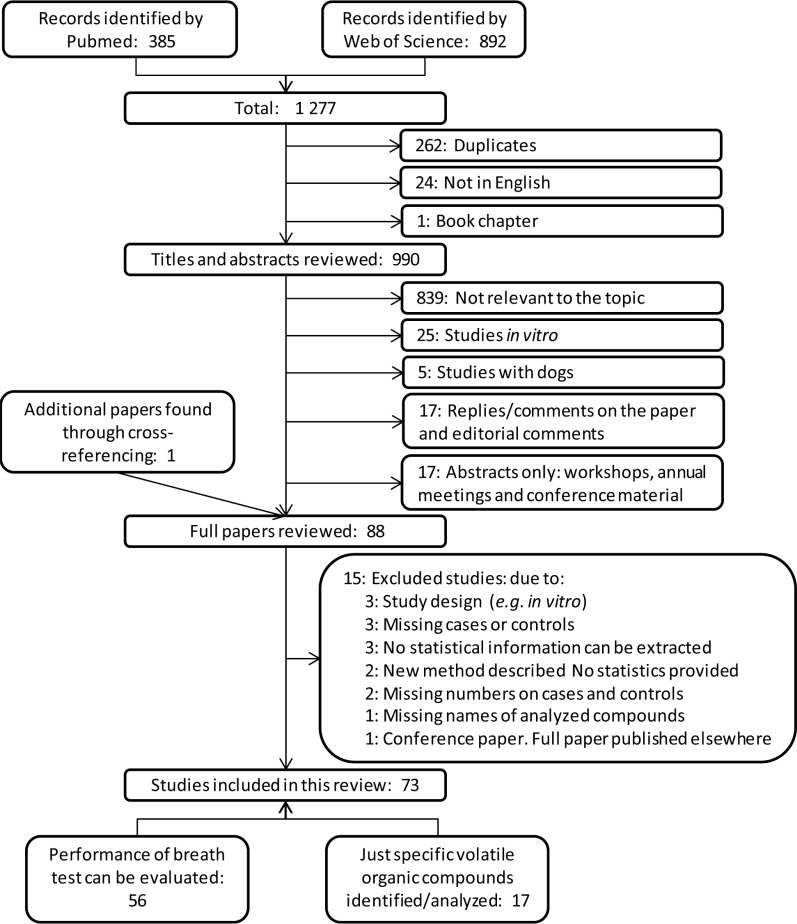
Flow diagram for literature search process Flow diagram for literature search process in Pubmed and Web of Science databases using following keywords: (cancer OR carcinoma OR adenocarcinoma OR tumor OR malignancy OR malignant disease) AND ((volatile AND (compound OR compounds OR marker OR markers OR biomarker OR biomarkers)) OR VOC OR VOCs OR breathprint OR breath-print OR breath print) AND (breath OR exhaled OR air).

Figure 1 shows the process of study selection. In total, 1277 papers were identified of which 262 were duplicates, 24 non-English papers and 1 book chapter. The remaining titles and abstracts were checked and studies not relevant to the topic were excluded. For 17 studies, no full paper could be accessed. Also, 15 full-text papers were excluded as some of the required information was missing (see Additional Figure S1).

In total, 73 studies met our inclusion criteria and were described in this review. The majority of them focused on malignancies in organs of the respiratory system (lung, *n* = 46; head and neck, *n* = 4) as these cancers have the biggest potential to be diagnosed by exhaled breath. Other malignancies also investigated with breath analysis included: breast cancer (*n* = 11), gastric cancer (*n* = 5), mesothelioma and colorectal cancer (each *n* = 3), cancers of the liver, thyroid, prostate and ovaries, and hematological and gynecological cancers (each *n* = 1).

### Design and methods

Study designs and methods of analysis are described in Table 1. To analyze exhaled breath most of the studies used gas chromatography-mass spectrometry (GC-MS, *n* = 42) and/or electronic nose (e-nose, *n* = 24). The most commonly used electronic noses were gold nanoparticles sensors-based e-nose [7] from the TECHNION group (8 studies) and commercially available Cyranose 320 [8] (six studies). Breath samples were stored in different containers with Tedlar bag being the most popular.

**Table 1:** Study characteristics: breath analysis technique, breath collection system or storage container and classifier

First author, year	Technique	Storage container	Classifier^a^
Studies which used electronic nose			
Di Natale, 2003 [9]	LibraNose	Sterile disposable bag	Partial least square DA
Chen, 2005 [74]	SAW sensors	Tedlar bag	Artificial neural networks
Machado, 2005 [75]	Cyranose 320	Mylar bag	Support vector machine
Mazzone, 2007 [76]	colorimetric sensors	No storing of samples	Random forest
Dragonieri, 2009 [77]	Cyranose 320	Tedlar bag	Linear canonical DA
D'Amico, 2010 [78]	QMS sensors	Tedlar bag	Partial least square DA
Shuster, 2011 [79]	NA-NOSE	No information	Support vector machine
Yu, 2011 [80]	MOS sensors	Tedlar bag	Principle component analysis
Chapman, 2012 [39]	Cyranose 320	Rapak bag	Linear canonical DA
Dragonieri, 2012 [26]	Cyranose 320	Tedlar bag	Canonical DA
Mazzone, 2012 [81]	colorimetric sensors	No storing of samples	Multinomial linear RA
Santonico, 2012 [82]	QMS sensors	Tedlar bag	Partial least square DA
Wang D, 2012 [83]	MOS-SAW sensors	Tedlar bag	Artificial neural networks
Broza, 2013 [11]^b^	NA-NOSE	Mylar bag	Discriminant factor analysis
Hubers, 2014 [16]	Cyranose 320	Tedlar bag	Multinomial linear RA
Leunis, 2014 [84]	DiagNose	Tedlar bag	Multinomial linear RA
McWilliams, 2015 [85]	Cyranose 320	Mylar bag	Discriminant factor analysis
Shehada, 2015 [86]	TPS-SiNW FET sensors	ORBO 420 Tenax TA sorption tubes	Discriminant factor analysis
Studies which used electronic nose and gas chromatography-mass spectrometry			
Hakim, 2011 [34]	NA-NOSE, SPME/GC-MS	Mylar bag	Support vector machine
Peled, 2012 [33]^b^	NA-NOSE, SPME/GC-MS	ORBO 420 Tenax TA sorption tubes	Discriminant factor analysis
Xu Z, 2013 [40]^b^	NA-NOSE, GC-MS	ORBO 420 Tenax TA sorption tubes	Discriminant factor analysis
Gruber, 2014 [87]	NA-NOSE, GC-MS	ORBO 420 Tenax TA sorption tubes	Discriminant factor analysis
Amal, 2015^OC^ [88]	NA-NOSE, GC-MS	ORBO 420 Tenax TA sorption tubes	Discriminant factor analysis
Amal, 2015^GC^ [18]	NA-NOSE, TD-GC-MS	ORBO 420 Tenax TA sorption tubes	Discriminant factor analysis
Studies which used gas chromatography-mass spectrometry			
Gordon, 1985 [28]	TD-GC-MS	Teflon sampling bag	Linear DA
Preti, 1988 [44]	TD-GC-MS	Tenax sorption tubes	-
Phillips, 1999 [89]^b^	TD-GC-MS	Portable electrical device^c^	DA
Phillips, 2003^BC^ [24]^b^	TD-GC-MS	Portable electrical device^c^	DA
Phillips, 2003^LC^ [66]^b^	TD-GC-MS	Portable electrical device^c^	DA
Poli, 2005 [10]	SPME/TD-GC-MS	bio-VOC breath sampler	Multinomial linear RA
Phillips, 2006 [23]^b^	TD-GC-MS	Portable electrical device^c^	Fuzzy logic
Phillips, 2007 [19]	TD-GC-MS	Portable electrical device^c^	Fuzzy logic
Phillips, 2008 [20]	TD-GC-MS	Portable electrical device^c^	Weighted digital analysis
Bajtarevic, 2009 [21]	SPME/GC-MS	Tedlar bag	-
Ligor, 2009 [22]	SPME/GC-MS	Tedlar bag	-
Peng, 2009 [90]	SPME/GC-MS	Mylar bag	-
de Genaro, 2010 [25]	TD-GC-MS	Tedlar bag	Discriminant factor analysis
Fuchs, 2010 [65]	SPME/GC-MS	Sealed headspace vial	-
Kischkel, 2010 [91]	SPME/GC-MS	Sealed headspace vial	-
Peng, 2010 [49]	SPME/GC-MS	Mylar bag	-
Phillips, 2010 [30]	TD-GC-MS	Portable electrical device^c^	Weighted digital analysis
Poli, 2010 [53]	SPME/GC-MS	bio-VOC breath sampler	DA
Qin, 2010 [27]	SPME/TD-GC-MS	Tedlar bag	Fisher's linear DA
Song, 2010 [29]	SPME/GC-MS	Tedlar bag	-
Patterson, 2011 [92]	TD-GC-MS	Teflon sampling bag	Linear DA, Quadratic DA, support vector machine
Rudnicka, 2011 [93]	SPME/GC-TOF-MS	Tedlar bag	Discriminant factor analysis
Ulanowska, 2011 [94]	SPME/GC-MS	Tedlar bag	DA
Buszewski, 2012 [15]	SPME/GC-MS	Tedlar bag	-
Mangler, 2012 [95]	TD-GC-MS	Tenax test tube	-
Wang Y, 2012 [13]	SPME/GC-MS	Tedlar bag	Linear DA
Amal, 2013 [14]	TD-GC-MS	ORBO 420 Tenax TA sorption tubes	-
Altomare, 2013 [96]	TD-GC-MS	Tedlar bag	Probabilistic neural networks
Filipiak, 2014 [12]	TD-GC-MS	Tedlar bag	-
Garcia, 2014 [97]	SPME/GC-MS	Tedlar bag	-
Li, 2014 [98]	SPME/GC-MS	Tedlar bag	Fisher's DA
Rudnicka, 2014 [32]	SPME/GC-MS	Tedlar bag	Artificial neural networks
Wang C, 2014^BC^ [99]	SPME/GC-MS	Glass vials	Partial least square DA
Wang C, 2014^CRC^ [100]	SPME/GC-MS	Glass vials	Partial least square DA
Zou, 2014 [41]	SPME/GC-MS	Tedlar bag	-
Guo, 2015 [101]	SPME/GC-MS	Glass vials	-
Studies which used other techniques			
Hietanen, 1994 [102]	Carbotrap/Carbosieve SIII-TD-GC	Vacu-sampler can	-
Rieder, 2001 [103]	PTR-MS	No storing of samples	-
Steeghs, 2007 [104]	PTR-MS	Tedlar bag	Logistic RA
Wehinger, 2007 [35]	PTR-MS	Tedlar bag	Fisher's quadratic DA
Westhoff, 2009 [31]	MCC/IMS	No storing of samples	Linear DA
Hauschild, 2012 [62]	MCC/IMS	No storing of samples	Random forest
Bousamra, 2014 [37]	FT-ICR-MS	Tedlar bag	Ruled
Fu, 2014 [36]	FT-ICR-MS	Tedlar bag	Ruled
Handa, 2014 [105]	MCC/IMS	No storing of samples	Decision Tree
Ma, 2014 [106]	SPME/GCxGC	Tedlar bag	-
Phillips, 2014 [38]^b^	GC-SAW	Portable electrical devicec	Weighted digital analysis
Xu H, 2014 [17]	MSPE	RTube collection system	-
Kumar, 2015 [107]	SIFT-MS	Nalophan	Logistic RA

a missing classifier identifies studies where no diagnostic performance of breath test was evaluated but concentrations of volatile compounds between cases and controls were compared

b patients attending the hospital with some complain were enrolled in the study and breath samples were collected before the final diagnosis

c portable electronic device [108]

^d^Rule - at least 2 out of 4 elevated VOCs present in breath

GC Gastric cancer

OC Ovarian cancer

LC Lung cancer

BC Breast cancer

CRC Colorectal cancer.

METHODS: GC – gas chromatography; MS – mass spectrometry; FT-ICR-MS – Fourier transform ion cyclotron resonance MS; GC-TOF-MS – GC-Time of flight-MS; MCC/IMS – multi capillary column-ion mobility spectrometry; MOS – metal oxide semiconductor; MSPE – magnetic solid-phase extraction; TD-GC-MS – thermal desorption-GC-MS; PTR-MS – proton transfer reaction-MS; QMS – quartz microbalance; SAW – surface acoustic wave; SIFT-MS – selected ion flow tube-MS; SPME – solid phase microextraction; TPS-SiNW-FET – trichloro-(phenethyl)silane-silicon nanowire-field effect transistor.

CLASIFIER: DA – discriminant analysis; RA – regression analysis.

The vast majority of studies were conducted in a case-control approach, in which clinically diagnosed patients were compared with controls without cancer. Eight studies enrolled patients coming to the hospital with some complaints for further investigations and breath samples were collected before the final diagnosis. Also, few studies investigated differences in exhaled breath composition before and after tumor resection [9-11] and VOCs released by cancer cells or tissues [12, 13]. Differences of volatiles between Caucasians and Chinese were investigated by Amal *et al*. [14]. Two more studies were conducted to compare the performance of exhaled breath analysis to the performance of canine detection [15] or DNA hypermethylation in sputum [16]. Despite the differences in studies designs, we focused and extracted information related just to breath analysis part in all of the studies.

### Study population

An overview on the studies and their population characteristics is shown in Additional Table S1. Studies were conducted in all parts of the world except South America and Africa. Numbers of people included into analyses varied from 14 (6 cases and 8 controls) [17] to 477 (99 cases and 378 controls) [18]. The majority of studies used healthy controls; however, a mixture of healthy controls and individuals with some medical conditions were used as a reference group in 9 studies and 8 studies exclusively used controls with benign medical conditions. Furthermore, same study populations were used in studies on lung cancer [19, 20] and [21, 22], breast cancer [23, 24] and mesothelioma [25, 26].

Further information on critical study design and data collection issues is presented in Additional Table S2. History of smoking is the main risk factor for lung cancer development; therefore, adjustment for smoking status between cases and controls is crucial. However, 8 studies on lung cancer did not provide information on smoking status at all. The majority of the studies collected alveolar breath, 12 studies focused on collecting maximum amount of exhaled breath (vital capacity) and 7 studies on collecting tidal breath. Also, around 25% of the studies did not perform lung washout or ambient air was not analyzed which might lead to exogenous (inhaled) compounds to be included into classification models. Time between breath collection and analysis was very short (analysis done immediately or within few hours) in most studies but extended up to six months in one study [18]. Although most of the studies included newly diagnosed untreated cancer patients, few studies recruited patients under different treatment regimens, and treatment might have had an influence on exhaled volatiles.

### Performance of classification models

Table 2 presents studies which reported sensitivity and specificity or other statistical information on classification of cancer cases and controls based on exhaled breath analysis. Only studies where classification based on pattern recognition by e-nose or modeling of multiple VOCs were included in this table (*n* = 48). Overall, numbers of VOCs included in the classification models varied from 3 [27-29] to 30 [20, 30].

**Table 2:** Breath test performance for cancer detection together with indication if values were corrected for overoptimism

First author, year	Cs (N)	Cn (N)	Sens	Spec	AUC	Acc	Corrected for overoptimism?^a^
Lung cancer								
Gordon, 1985 [28]	12	9	-	-	-	93.0	NO-model on selected 3 VOCs
	12	9	100.0	100.0	-	100.0	NO-model on selected 22 VOCs
Phillips, 1999 [89]	60	48	71.7	66.7	-	69.4	YES-LOOCV
Phillips, 2003 [66]	67	41	85.1	80.5	-	83.3	YES-LOOCV
	-	91^b^	-	37.4	-	-	YES-validation set
Chen, 2005 [74]	5	5	80.0	80.0	-	80.0	YES-validation set
Machado, 2005 [75]	14	62	71.4	91.9	-	88.2	YES-validation set
Poli, 2005 [10]	36	110	72.2	93.6	-	88.4	NO-model on selected VOCs
Mazzone, 2007 [76]	49	94	73.3	72.4	-	-	YES-RSS-70:30%
Phillips, 2007 [19]	193	211	84.6	80.0	0.88	-	YES-RSS-2:1
Steeghs, 2007 [104]	11	57	-	-	0.81	-	NO-model on selected VOCs
Wehinger, 2007 [35]	17	170	54.0	99.0	-	96.0	YES-average of 1.000 RSS-60:40%
Phillips, 2008 [20]	193	211	-	-	0.87	-	NO-VOCs preselected, then RSS
Bajtarevic, 2009 [21]	65	31	52.0	100.0	-	-	NO-model on selected 4 VOCs
	65	31	71.0	100.0	-	-	NO-model on selected 15 VOCs
	65	31	80.0	100.0	-	-	NO-model on selected 21 VOCs
Dragonieri, 2009 [77]	10	10	-	-	-	90.0	YES-cross-validation
	10	10^c^	-	-	-	85.0	
Ligor, 2009 [22]	65	31	51.0	100.0	-	-	NO-model on selected 8 VOCs
Westhoff, 2009 [31]	32	54	100.0	100.0	-	100.0	NO-first VOCs selected, then LOOCV
D'Amico, 2010 [78]	28	36	85.0	100.0	-	93.8	YES-LOOCV
	28	28^d^	92.8	78.6	-	85.7	
Poli, 2010 [53]	40	38	90.0	92.1	-	91.0	YES-LOOCV
Hakim, 2011 [34]	20	26	100.0	92.3	-	95.7	YES-average of all sample splits
Yu, 2011 [80]	9	9	100.0	88.9	-	94.4	NO-model on selected peaks
Mazzone, 2012 [81]	83e	137	-	-	0.701	-	NO-model on selected sensor parameters
	9f	137	-	-	0.8	-	
Peled, 2012 [33]	50	19	86.0	96.0	0.986	88.0	YES-LOOCV
Santonico, 2012 [82]	20	10	85.0	85.0	-	85.0	YES-LOOCV
Wang D, 2012 [83]	47	42	93.6	83.4	-	88.8	YES-LOOCV
Wang Y, 2012 [13]	85	158	96.5	97.5	-	97.1	YES-LOOCV
Broza, 2013 [11]	12	5	100.0	80.0		94.1	YES-LOOCV
Bousamra, 2014 [37]	107	40	87.9	77.5	-	85.0	YES-≥2 out of 4 elevated VOCs present (VOCs selected on the different population)
Fu, 2014 [36]	97	32	92.8	81.3	-	89.9	NO-≥2 out of 4 elevated VOCs present
Handa, 2014 [105]	50	39	76.0	100.0	-	-	NO-model on selected 10 VOCs
Hubers, 2014 [16]	18	8	94.4	12.5	-	69.2	YES-validation set
Rudnicka, 2014 [32]	108	121	74.0	73.0	0.97	-	YES-RSS-50:25:25%
McWilliams, 2015 [85]	25	166	-	-	0.803	-	YES-average of 10 RSS-2:1
Breast cancer								
Phillips, 2003 [24]	51	42	88.2	73.8	-	81.7	YES-LOOCV
	51	50^g^	60.8	82.0	-	71.3	
Phillips, 2006 [23]	51	42	93.8	84.6	0.9	-	YES-RSS-70:30%
	-	50^g^	-	32.0	-	-	YES-validation set
Phillips, 2010 [30]	54	204	75.3	84.8	0.83	-	YES-10 RSS-2:1
Patterson, 2011 [92]	20	20	72.0	64.0	-	77.0	YES-average of 10.000 RSS-60:40%
Li, 2014 [98]	22	24	68.2	91.7	-	80.4	YES-LOOCV
Phillips, 2014 [38]	35	93	-	-	0.73	-	YES-LOOCV
	35	79^g^	-	-	0.67	-	
Colorectal^CRC^, gastric^GC^, ovarian^OC^, liver^LVC,^ head and neck^HNC^ cancer and malignant mesothelioma^MM^							
Qin, 2010^LVC^ [27]	30	36	83.3	91.7	-	87.9	NO-first 3 VOCs selected, then LOOCV
	-	27^h^	-	66.7	-	-	
Hakim, 2011^HNC^ [34]	16	26	100.0	92.3	-	95.2	YES-average of all sample splits
Chapman, 2012^MM^ [39]	20	42	90.0	91.0	-	90.5	YES-RSS (10 Cs and 32 Cn for validation)
	-	18^d^	-	83.3	-	-	YES-validation set
Dragonieri, 2012^MM^ [26]	13	13	-	-	0.893	84.6	YES-LOOCV
	13	13^i^	-	-	0.917	80.8	
Altomare, 2013^CRC^ [96]	15	10	80.0	70.0	-	76.0	YES-validation set
Xu Z, 2013GC [40]	37	93	89.0	90.0	-	90.0	YES-RSS-75:25%
Gruber, 2014^HNC^ [87]	22	19	77.0	90.0	-	83.0	YES-LOOCV
	22	21^j^	77.0	90.0	-	84.0	
Leunis, 2014^HNC^ [84]	36	23	-	-	0.85	-	YES-bootstrapped value
Amal, 2015^OC^ [88]	48	48	78.6	100.0	-	89.3	YES-RSS-70:30%
	48	86^k^	57.1	59.0	-	58.0	
	48	134^l^	71.4	71.8	-	71.7	
Amal, 2015^GC^ [18]	99	325^m^	73.3	97.9	-	92.0	YES-RSS-70:30%
	99	53^n^	86.7	86.7	-	86.7	
Kumar, 2015^GC^ [107]	81	121	86.7	81.2	0.87	-	YES-average of 10 RSS-2:1
Shehada, 2015^GC^ [86]	30	77	71.0	89.0	-	85.0	YES-RSS-75:25%

Cn - cases; Cs - controls, N - number of cases/controls; Sens - sensitivity; Spec - specificity; AUC - area under the receiver operating characteristic curve; RSS - random sample split-training set size: testing set size: validation set size. Numbers of cases and controls are total study population size and performance of breath test corresponds to testing (validation) set; LOOCV - leave-one-out cross-validation; VOCs-volatile organic compounds.

a NO indicates studies which used same study population for model building and testing

b abnormal X-rays, no cancer

c Chronic obstructive pulmonary disease

d lung diseases

^e^non-small cell lung cancer

^f^small cell lung cancer

g abnormal mammography

h hepatoccirosis

i exposed to asbestos

j benign head and neck conditions

k ovarian benign conditions

l healthy+ovarian benign conditions

m Operative link on gastric intestinal metaplasia assessment stage 0-IV;

n gastric ulcer.

For lung cancer reported sensitivity (specificity) varied from 51% (13%) to 100% (100%). However, rigorous validation was not performed in all of the studies, which may have led to overoptimistic results. This particularly applies to two studies that reported perfect discrimination between cases and controls, with 100% overall accuracy [28, 31]. Nevertheless, very good diagnostic performance was also reported in studies with validation showing AUCs of 0.97 [32] and 0.986 [33], or overall accuracy of 96% [34, 35]. Two studies applied a different classification rule and classified participants “positive” when 2 or more out of 4 VOCs had higher concentrations than the set cut-offs and found relatively high levels of accuracies of 90% [36] and 85% [37]. Also, two classification models on the same study population showed exactly same performance while including 4 [21] and 8 [22] VOCs. Both classification models included the same three VOCs (1-propanol, 2-butanone, benzaldehyde), which were not used together by any other study.

Six studies reported the diagnostic performance of breath test for distinguishing breast cancer patients and healthy controls. The best discriminatory performance was achieved by Phillips *et al*. in 2006 [23], who reported AUC of 0.9. On the other hand, the same model validated in women with abnormal mammography findings showed specificity as low as 32%. Other studies by the same authors also showed better performance of the classification models when comparing cancer cases to healthy women rather than to women with abnormal mammography findings [24, 38].

Good diagnostic performance was also reported in most of the studies focusing on the cancer organs other than lung and breast, and AUC or accuracy of 0.9 or higher was reported in studies on head and neck cancer [34], malignant mesothelioma [39], and gastric cancer [18, 40].

### Performance of individual VOCs

The performance of classification using individual VOCs as cancer biomarkers in exhaled breath was reported in 8 studies and is presented in Table 3. Several volatiles, i.e., 3-hydroxybutan-2-one, showed promising results for different cancer sites. One study validated its results in a different population sample and showed superb performance of hexadecanal (AUC=1.00) [41]. Volatile organic compounds which were used to build a classification model or whose concentrations were significantly different between cancer cases and controls in at least three independent studies are presented in Additional Table S3. Ethenylbenzene (styrene), heptanal and nonanal were the most commonly described compounds (each in 9 independent studies). Interestingly, these studies were performed on different cancer types. By contrast, 1-propanol was described just by the studies on lung cancer, 3 studies showed significantly different concentrations in exhaled breath and 4 others included this compound in classification models.

**Table 3:** Performance of the individual compounds together with the concentration gradient in the cancer patients

First author, year	Cancer site	Volatile compound	Cut-off	Sens	Spec	AUC	Gradient
Fuchs, 2010 [65]	lung	pentanal	0.275 nmol/L	75.0	95.8	-	up
		hexanal	1.208 nmol/L	8.3	91.7	-	up
		octanal	1.068 nmol/L	58.3	91.7	-	up
		nonanal	8.433 nmol/L	33.3	95.8	-	up
Song, 2010 [29]	lung	butan-1-ol	3.67 ng/L	95.3	85.4	0.94	up
		3-hydroxybutan-2-one	3.81 ng/L	93.0	92.7	0.96	up
Wang Y, 2012 [13]	lung	hexadecanal	?	96.5	89.2	0.949	-
Handa, 2014 [105]	lung	dodecane	?	70.0	89.7	-	up
Zou, 2014 [41]^a^	lung	5-(2-methylpropyl)nonane	?	-	-	0.845	up
		2,6-di-tert-butyl-4-methylphenol	?	-	-	0.724	up
		2,6,11-trimethyldodecane	?	-	-	0.846	up
		hexadecanal	?	-	-	1.00	up
		8-hexylpentadecane	?	-	-	0.672	up
Mangler, 2012 [95]	breast	3-methylhexane	−0.55 μg/m³	100.0	40.0	-	down
		dec-1-ene	−0.125 μg/m³	100.0	40.0	-	down
		Caryophyllene^b^	−0.05 μg/m³	100.0	60.0	-	down
		naphthalene	0.05 μg/m³	90.0	70.0	-	down
		trichloroethene	0.05 μg/m³	80.0	70.0	-	up
Li, 2014 [98]	breast	hexanal	10.32 ppbv	77.3	79.2	0.79	up
		heptanal	9.98 ppbv	68.2	91.7	0.823	up
		octanal	12.9 ppbv	63.6	87.5	0.734	up
		nonanal	23.14 ppbv	72.7	95.8	0.832	up
Qin, 2010 [27]^c^	liver	3-hydroxybutan-2-one	2.44 ng/L	83.3	91.7	0.926	up
		ethenylbenzene	14.92 ng/L	66.7	94.4	0.812	up
		decane	1.64 ng/L	86.7	58.3	0.798	up

Sens - sensitivity; Spec - specificity; AUC - area under the receiver operating characteristic curve; ppbv - parts per billion by volume.

a performance in the validation set

b 4,11,11-trimethyl-8-methylidenebicyclo[7.2.0]undec-4-ene

c comparison between liver cancer patients and healthy controls.

## DISCUSSION

In this paper, we present a comprehensive up to date overview of studies on diagnostic performance of VOCs in cancer detection. Our review identified 73 studies which used breath analysis for classification of cancer cases and controls or analyzed specific VOCs in exhaled breath of cancer patients and healthy individuals. The majority of the studies focused on lung cancer; however, recent reports addressed other common malignancies including breast, gastric and other types of cancer. Very good diagnostic performance of breath tests was achieved, but one out of four studies lacked appropriate correction for overoptimism. It is worth pointing out, that studies differed significantly with respect to breath analysis techniques and data analysis methods. Based on current evidence, VOCs seem to hold a great potential in cancer diagnostics; nevertheless, the ultimate role of these markers for cancer screening needs to be determined and established in large scale studies conducted in true screening setting.

Breath analysis is a young field of research and majority of the studies were performed in recent years. That hundreds of VOCs are present in human breath is known for decades [42]. In the 1980s, the first studies reported higher levels of some volatiles in the breath of lung cancer patients [28, 43, 44] and these studies fostered substantial interest in research of cancer specific biomarkers in breath. The first studies focused on identifying specific volatile organic compounds for diseases of interest using methods such as GC-MS, which is expensive, time-consuming and requires well-trained personnel for performing sample collection and analysis. Furthermore, identification of detected compounds is not straight forward and reference libraries have to be checked and validated using mass and retention time of the known standards. The latter, among other reasons, led researchers to look for different methods to analyze exhaled breath, one such technology is the nanomaterial-based sensor arrays that could be a good solution for solving the problems mentioned above [45-47].

As the main difference from standard analytic techniques, electronic nose mimics mammalian olfaction [48] and in that it cannot distinguish specific VOCs but is based on pattern recognition. First, the e-nose needs to be trained to build a database for recognition, and then it can be applied for classifying other unknown samples. The crucial factors of meaningful pattern recognition are the size of the training set and how good these samples represent tested populations. As one way to improve the performance of e-nose, a combination between other techniques and e-nose is possible, i.e. specific VOCs can be identified by GC-MS and used to select sensors most sensitive to target compounds [49].

Independent of analysis techniques, breath sample collection and storage are major challenges in breath research studies. The stability of compounds in different bags have been investigated [50-52], which showed that some polar compounds, including water, diffuse rather quickly through Tedlar bag walls, while other compounds are quite stable. Aldehydes were shown to be rather stable in Bio-VOC sampler in the first 10 hours after collection while analysis was done in less than 2 hours [53]. Sample storage time recorded in this review was very short and those five studies which exceeded few months for storage, used thermal sorption tubes which are suitable for long-term storing [54]. Apart from loss of compounds due to diffusion through the bags' walls, some compounds might be released by the bags material and accumulate in the collected air sample [55]. Reusing the same bag might represent another challenge as flushing and heating failed to remove some of the compounds from Tedlar bag [56]. Finally, concentration of VOCs also strongly depends on breath collection method. Alveolar breath has higher levels of exhaled components than the whole breath without separation [57, 58] and also the lowest concentrations of contaminants [59]. Standardization of the breath collection process appears crucial for further advances in breath-based biomarker research. Additionally to ambient air analysis or lung washout before breath sampling [60, 61], other standardization processes including recommendations for sample storage in thermal desorption tubes or ways to avoid some confounders while recruiting hospital personnel rather than healthy individuals outside the hospital were recently suggested as well [54].

A key issue in the analysis of high dimensional data such as those obtained from breath analysis is rigorous control for overoptimism by internal or external validation. External validation is particularly interesting where performance of classification model can be demonstrated on different populations or different recruitment conditions, as the purpose of marker discovery studies is their potential application in future screening strategies. Replication of the results might not be easily achieved as different methods and analysis techniques are being used by different research groups. Furthermore, different results were achieved even in the same study while applying different computational approaches for data analysis [25, 62]. Still, internal validation by performing, for example, random sample split or leave-one-out cross-validation can help to get as close to the real estimate as it can get but does not guarantee good performance on different study populations.

Adjusting for covariates when building a classification model for breath analysis is another challenge as it still remains unclear which covariates should be taken into account. Controversial results were shown for the impact of age, gender and smoking status on VOCs [31, 63-66] for standard analysis techniques. On the other hand, results of “breathprints” pattern analysis with e-nose showed to be insensitive to various covariates including the ones mentioned above [34, 49], but it remains unclear and requires further research what factors may confound study results using electronic nose. While matching or adjusting for covariates is crucial for evaluating the discriminatory potential of VOCs *per se*, combined use of VOCs and covariates may provide the most powerful discriminatory algorithms for screening practice.

To date, there is no “universal” tumor marker that can detect any type of cancer; however, development of the VOCs field could potentially provide a tool for unified technological approaches in cancer screening. So far, the set of identified VOC patterns varies considerably among the studies. Even though promising results have been reported for certain single markers in individual studies (i.e. hexadecanal), enhanced accuracy for classification of cancer cases and controls is likely to be achieved by the combination of several compounds. Furthermore, the same compound may not be specific for a certain cancer but it might be characteristic for several types of cancer. For example, formaldehyde (methanal) was suggested to be a potential biomarker for breast [67], prostate and bladder cancers [68]. At the same time, there is emerging evidence for cancer specific markers. A review on potential cancer specific compounds was published recently [5] in which metabolic pathways for volatiles arising from bodily fluids was explained, and furthermore the potential of these compounds to be biomarkers for cancer was discussed.

Breath analysis as a cancer detection method and potential VOCs biomarkers for cancer were previously discussed and summarized [5, 69]. Queralto *et al*. covered the existing evidence on exhaled breath analysis and cancer detection [70], but provided only a brief description of the results and focused mainly on differences between array-based sensors. Recently, increasing interest has been devoted to novel instruments for breath analysis. Reviews were published on different electronic noses used until then for biomedical and other applications [71], advances in breath analysis using e-nose for detection of various diseases [72] and nanosensor technologies used for VOCs detection [45, 73].

Differently to previous reviews, we extensively discuss key shortcomings of methodological issues, such as correction for overoptimism, performance of the validation studies and influence of potential confounders. Furthermore, we did not restrict this systematic review to specific cancer site or analysis method, as we wanted to understand exact potential of application of breath analysis to cancer detection at this stage. Nevertheless, our review has certain limitations that need to be acknowledged. Despite a comprehensive research in two independent databases we cannot exclude the possibility of having missed relevant studies. Standardized summarization and presentation of results was hampered by heterogeneity in the reporting in the original studies. We did not include *in vitro* studies because performance of these markers may not always translate into direct clinical applications in screening and diagnostic settings. We also did not include studies which used sniffer dogs, as potential implementation of canine-based diagnosis in health care settings might face logistic limitations.

In conclusion, breath analysis is a young field of research with great potential in cancer screening. For establishing an accurate test in a point of care screening setting, a large throughput sampling protocol of participants is required, i.e., collection and analysis time should be short, the method itself should be cheap, non-invasive, and with minimal health risk. In order to foster implementation in practice, larger studies should be implemented in true screening settings, paying particular attention to standardization in breath collection, consideration of covariates, adjustment for overoptimism, and validation in independent population samples. With further advancements in the area, breath test may have the potential to become a useful supplement and improve existing screening tools for a variety of cancers.

## MATERIALS AND METHODS

A systematic literature search was performed in literature published until April 30, 2015 by searching Pubmed and Web of Science databases using the following combination of keywords: (cancer OR carcinoma OR adenocarcinoma OR tumor OR malignancy OR malignant disease) AND ((volatile AND (compound OR compounds OR marker OR markers OR biomarker OR biomarkers)) OR VOC OR VOCs OR breathprint OR breath-print OR breath print) AND (breath OR exhaled OR air). Full-text original studies in English language which reported statistics on discrimination between cancer cases and controls, or studies which investigated specific VOCs, were included in this systematic review. In addition, reference lists were checked for relevant published studies for inclusion. Studies exclusively conducted *in vitro* or with sniffer dogs were not considered in this review.

Data extraction was carried out independently by two of the authors, AK and JAH, and included characteristics of study populations, such as numbers of cases and controls, their age, sex and smoking prevalence, as well as the country where study populations were recruited. Study design and methods used for breath analysis were also recorded. Indicators for diagnostic value were extracted both for individual VOCs as well as for multi-VOCs classifiers where provided. The following statistical parameters were considered: sensitivity and specificity, accuracy and area under the receiver operating characteristic curve (AUC). Correction for overoptimism and validation was recorded for each study. The most reliable information was considered to avoid overoptimism, i.e. bootstrapped or cross-validated values were extracted wherever such results were provided. For studies which used random sample split to create a model and validate it separately, values corresponding to validation set were considered.

Additionally, we extracted names of VOCs which showed a significantly different concentration in exhaled breath from cancer cases and controls, or which were used to build a classification model. The IUPAC name [6] was checked for all extracted compounds to detect synonyms and to ensure comparable results.

Missing information in the tables was calculated where possible, i.e., accuracy was assessed as the sum of correctly classified cases and controls divided by total number of people in a classification model. Also, when comparison of exhaled breath was made just between two groups (cases and controls) but authors provided a study population description for separate smaller sub-groups, then numbers were added up or weighted averages (e.g. of age) were calculated where possible.

Additionally, some quality criteria were checked for the studies and included in this systematic review. Comparability with respect to gender (or smoking status) was determined by the difference in percent units between proportion of males (or smokers) between cancer cases and controls. As for age, difference among median (or mean) ages between study groups was calculated. Comparability with respect to these variables was set to “yes” if the difference was not greater than 10 units and “no” otherwise. Other potentially important information for evaluating and comparing the results between studies was extracted, such as collection time and which breath part was used for analysis, analysis time, restrictions, potential preceding treatment of cancer cases, and exclusion criteria used when recruiting patients in each of the studies.

## SUPPLEMENTARY MATERIAL FIGURE AND TABLES


