# Supplementary material for: Detection of cancer through exhaled breath: a systematic review
Source: Oncotarget. 2015 Sep 30;6(36):38643–57. doi: 10.18632/oncotarget.5938 (PMC4770726; doi:10.18632/oncotarget.5938)
Supplement: Supplementary file 1 [file oncotarget-06-38643-s001.pdf]

Detection of cancer through exhaled breath: a systematic review

Supplementary Material

Figure S1. Reasons for excluding studies from this systematic review.

Study design: 1–Study with mice and then results were tested on people (3 cases and 3 controls); 2–Study with stomach tissue and then results were tested on people (3 cases and 10 controls); 3–Study with simulated data, not real samples

1. Ebeler SE, Clifford AJ, Shibamoto T. Quantitative analysis by gas chromatography of volatile carbonyl compounds in expired air from mice and human. J Chromatogr B Biomed Sci Appl. 1997;702(1-2):211-5.
2. Ligor T, Szeliga J, Jackowski M, Buszewski B. Preliminary study of volatile organic compounds from breath and stomach tissue by means of solid phase microextraction and gas chromatography-mass spectrometry. J Breath Res. 2007;1(1):016001.
3. Peng G, Trock E, Haick H. Detecting simulated patterns of lung cancer biomarkers by random network of single-walled carbon nanotubes coated with nonpolymeric organic materials. Nano Lett. 2008;8(11):3631-5.

New method described: no statistical information about performance of the method

4. Na N, Liu H, Han J, Han F, Liu H, Ouyang J. Plasma-Assisted Cataluminescence Sensor Array for Gaseous Hydrocarbons Discrimination. Analytical Chemistry. 2012;84(11):4830-6.
5. Zhang G, Guo X, Wang S, Wang X, Zhou Y, Xu H. New graphene fiber coating for volatile organic compounds analysis. J Chromatogr B Analyt Technol Biomed Life Sci. 2014;969:128-31.

Missing required information: 6–No cancer cases; 7, 8–No controls; 9, 10–Missing information on number of cancer cases and controls

6. Silva LIB, Freitas AC, Rocha-Santos TAP, Pereira ME, Duarte AC. Breath analysis by optical fiber sensor for the determination of exhaled organic compounds with a view to diagnostics. Talanta. 2011;83(5):1586-94.
7. Chatterjee S, Castro M, Feller JF. An e-nose made of carbon nanotube based quantum resistive sensors for the detection of eighteen polar/nonpolar VOC biomarkers of lung cancer. Journal of Materials Chemistry B. 2013;1(36):4563-75.
8. Hou C, Lei J, Huo D, Song K, Li J, Luo X, et al. Discrimination of Lung Cancer Related Volatile Organic Compounds with a Colorimetric Sensor Array. Analytical Letters. 2013;46(13):2048-59.
9. Kumar S, Huang J, Abbassi-Ghadi N, Spanel P, Smith D, Hanna GB. Selected ion flow tube mass spectrometry analysis of exhaled breath for volatile organic compound profiling of esophago-gastric cancer. Anal Chem. 2013;85(12):6121-8.
10. Wu Y, Huo D, Hou C, Fa H, Yang M, Luo X. Colorimetric Artificial Nose for Identification of Breath Volatile Organic Compounds of Patients with Lung Cancer. Chemical Research in Chinese Universities. 2014;30(4):572-7.

No statistical information provided: 11–Visual representation of results, some cases were excluded without explanation; 12–Concentrations of volatile organic compounds were measured, but no classification rule provided; 13–different sensors parameters were tested, but no overall classification into cases and controls was done

11. Gaspar EM, Lucena AF, Duro da Costa J, Chaves das Neves H. Organic metabolites in exhaled human breath--a multivariate approach for identification of biomarkers in lung disorders. J Chromatogr A. 2009;1216(14):2749-56.
12. Yu H, Xu L, Wang P. Solid phase microextraction for analysis of alkanes and aromatic hydrocarbons in human breath. Journal of Chromatography B-Analytical Technologies in the Biomedical and Life Sciences. 2005;826(1-2):69-74.
13. Tran VH, Chan HP, Thurston M, Jackson P, Lewis C, Yates D, et al. Breath Analysis of Lung Cancer Patients Using an Electronic Nose Detection System. Ieee Sensors Journal. 2010;10(9):1514-8.

Other reasons: 14–Proton transfer reaction-mass spectrometry was used, but masses were not identified; 15–Conference paper, full paper published elsewhere

14. Schmutzhard J, Rieder J, Deibl M, Schwentner IM, Schmid S, Lirk P, et al. Pilot study: volatile organic compounds as a diagnostic marker for head and neck tumors. Head Neck. 2008;30(6):743-9.
15. Chen X, Cao M, Hao Y, Li Y, Wang P, Ying K, et al. A Non-invasive detection of lung cancer combined virtual gas sensors array with imaging recognition technique. Conf Proc IEEE Eng Med Biol Soc. 2005;6:5873-6.

Table S1. Study population characteristics by cancer site: country where participants were recruited, number of male (M) and female (F) participants, age (Mean±s.d. OR median(range)) and smoking status (current/former/non-smoker) for cases (Cs) and controls (Cn).

First author, year	Country	Cases			Controls			Comments
		M/F	Age	Smoking	M/F	Age	Smoking	
**Lung cancer**								
Gordon, 1985 [1]	US	11/1	61±10	?/?/0	8/1	?(25-70)	2/?/?	
Preti, 1988 [2]	US	7/3	66±7	4/6/0	4/4	60±4	4/1/3	Cn: age-matched
					1/7	28±6	3/1/4	Cn: younger
Phillips, 1999 [3]	US	34/26	67±13	?/?/5	29/19	61±13	?/?/12	Cn: abnormal chest x-rays
Di Natale, 2003 [4]	IT	35	?	?	18	?	?	
		9	?	?				Cs: after surgery
Phillips, 2003 [5]	US/UK	48/19	68±10	?/?/3	16/25	70±13	?/?/18	Cn: healthy
					41/50	58±14	?	Cn: abnormal chest x-rays
Chen, 2005 [6]	CN	20	?	?	22	?	?	Training set
		5	?	?	5	?	?	Validation set
Machado, 2005 [7]	US	10/4	64±3	2/12/0	18/27	45[WA]	6/17/22	Training set
		10/4	61±13	?	31/31	45[WA]	?/?/≥30	Validation set
		28/8	67(?)	2/28/6	27/23	56(?)	0/0/50	Cn: non-smokers
Poli, 2005 [8]	IT				30/5	54(?)	35/0/0	Cn: smokers
					18/7	70(?)	1/21/3	Cn: COPD
Mazzone, 2007 [9]	US	24/25	65±?	13/35/1	44/50	56[WA]	5/48/41	
Phillips, 2007 [10]	US	96/97	66±11	33/134/24	106/105	68±6	78/133/0	
Steeghs, 2007 [11]	ND	11	56±5	11/0/0	57/0	60±5	57/0/0	
Wehinger, 2007 [12]	AT	13/4	62±11	9/5/4	83/87	41±13	60/11/95	
Phillips, 2008 [13]	The same as in Phillips, 2007							
Bajtarevic, 2009 [14]	AT	41/24	63(37-84)	28/31/6	15/16	38(21-87)	7/2/22	
Dragonieri, 2009 [15]	ND	10/0	66±9	2/7/1	4/6	58±8	0/0/10	Cn: healthy
					8/2	61±6	6/4/0	Cn: COPD
Ligor, 2009 [16]	The same as in Bajtarevic, 2009							
Peng, 2009 [17]	IL	40	?(28-60)	0/15/25	56	?(28-60)	17/0/39	
Westhoff, 2009 [18]	DE	24/8	65±10	7/17/6	39/15	46±12	12/0/42	
D'Amico, 2010 [19]	IT	28	62±6	0/17/11	36	?(50-70)	0/0/36	Cn: healthy
					28	61±7	0/17/11	Cn: lung diseases
Fuchs, 2010 [20]	DE	11/1	68±9	0/12/0	10/14	36±12	12/0/12	
Kischkel, 2010 [21]	DE	23/8	68±?	0/29/2	19/43	34[WA]	35/0/27	
Peng, 2010 [22]	IL	30	?	12/5/8	22	?	4/0/17	
Poli, 2010 [23]	IT	28/12	68±10	21/12/7	17/21	49±15	0/10/28	
Song, 2010 [24]	CN	34/9	58±8	0/21/22	34/7	48±7	0/0/41	
Hakim, 2011 [25]	IL	22/3	66±8	?	17/23	45±13	?	
Yu, 2011 [26]	CN	9	?	7/?/?	9	?	5/?/?	
Buszewski, 2012 [27]	PL	29	?	?	44	?	?	
Hauschild, 2012 [28]	DE	54	?	?	35	?	?	
Rudnicka, 2011 [29]	PL	17/6	?(51-78)	21/0/2	10/20	36[WA]	6/?/?	
Ulanowska, 2011 [30]	PL	99/38	?(38-86)	?	40/103	?(20-58)	41/0/102	
Mazzone, 2012 [31]	US	49/43	69±?	25/58/9	65/72	59±?	28/71/35	
Peled, 2012 [32]	US	31/22	65±7	19/26/8	15/4	61±7	6/11/2	Cn: benign conditions
Santonico, 2012 [33]	IT	16/4	67±8	8/7/5	8/2	65±8	3/4/3	Cn: benign conditions
Wang D, 2012 [34]	CN	35/12	?(32-80)	24/0/23	14/28	?(30-75)	6/0/36	

Table continued on following page

First author, year	Coun try	Cases			Controls			Comments
		M/F	Age	Smoking	M/F	Age	Smoking	
Wang Y, 2012 [35]	CN	24/61	62±10	47/0/38	36/52	45±9	29/0/59	Cn: healthy
					26/44	54±13	23/0/47	Cn: benign conditions
					62/96	49^{WA}	52/0/106	Cn: all
Broza, 2013 [36]	IL	7/5	65±7	5/5/2	3/2	64±7	2/2/1	Cn: benign conditions
Bousamra, 2014 [37]	US	107	66±10	44/51/12	40	51±15	12/14/14	Cn: benign conditions
Filipiak, 2014 [38]	AT	25/11	63±7	0/?/?	12/16	52±17	0/?/?	
Fu, 2014 [39]	US	97	?	?	32	?	?	Cn: benign conditions
Handa, 2014 [40]	JP	31/19	68±10	?/?/17	25/14	32±8	?/?/32	
Hubers, 2014 [41]	ND	12/8	65±8	7/12/0	22/9	65±9	12/19/0	Training set
		10/8	64±8	10/8/0	4/4	53±7	4/4/0	Validation set
Ma, 2014 [42]	CN	10/3	63±7	5/0/8	16/9	35^{WA}	8/0/17	
Rudnicka, 2014 [43]	PL	76/31	51	?	31/90	49^{WA}	?	
Xu H, 2014 [44]	CN	6	?	?	8	?	?	
Zou, 2014 [45]	CN	58/21	63±8	15/40/24	63/29	57^{WA}	21/45/26	Training set
		58	?	?	20	?	?	Validation set
McWilliams, 2015 [46]	CA	12/13	67±6	9/16/0	86/80	63±7	87/79/0	
**Breast cancer**								
Hietanen, 2013 [47]	UK	0/20	61(37-85)	?	?	?	?	Cn: benign conditions
Phillips, 2003 [48]	US	0/51	61±12	12/6/31	0/42	64±18	3/11/20	Cn: healthy
					0/50	?	?	Cn: abnormal mammogram
Phillips, 2006 [49]	The same as in Phillips, 2003							
Peng, 2010 [22]	IL	0/22	?	1/1/19	22	?	4/0/17	
Phillips, 2010 [50]	US	0/54	55±7	2/0/38	0/204	55±11	26/0/177	
Patterson, 2011 [51]	US	0/20	53±?	?	0/20	55±?	?	
Shuster, 2011 [52]	IL	0/13	?	3/1/9	0/7	?	2/0/5	Cn: healthy
					0/16	?	4/0/10	Cn: cancer precursors
Mangler, 2012 [53]	DE	0/10	65±?	?	0/10	59±?	?	
Li, 2014 [54]	CN	0/22	?	0/0/22	0/24	?	0/0/24	
Phillips, 2014 [55]	US/ND	0/35	?	?	0/93	?	?	Cn: healthy
					0/79	?	?	Cn: abnormal mammogram
Wang C, 2014 [56]	CN	0/39	53±11	2/?/?	0/91	42^{WA}	1/?/?	
**Other cancers (colorectal^{CRC}, gastric^{GC}, ovarian^{OC}, liver^{LVC}, prostate^{PC}, thyroid^{TC}, head and neck^{HNC} and heamatological and gynecological^{HGC} cancers and malignant mesothelioma^{MM})**								
^{HGC}Rieder, 2001 [57]	AT	16	?	?	100	?	?	
^{LVC}Qin, 2010 [58]	CN	26/4	53±12	8/0/22	24/12	49±11	7/0/29	Cn: healthy
					18/9	52±11	3/2/22	Cn: hepatocirrhosis
^{HNC}Hakim, 2011 [25]	IL	19/3	60±9	?	17/23	45±13	?	
^{MM}Chapman, 2012 [59]	AU	18/2	69±10	0/12/8	34/8	67±14	0/12/30	Cn: healthy
					18/0	71^{WA}	0/12/6	Cn: lung diseases
^{MM}de Genaro, 2010 [60]	IT	11/2	61±12	0/5/8	5/8	52±16	0/0/13	Cn: healthy
					9/4	67±10	0/4/9	Cn: exposed to asbestos
^{CRC}Peng, 2010 [22]	IL	26	?	10/5/11	22	?	4/0/17	
^{PC}Peng, 2010 [22]	IL	18	?	3/3/12	22	?	4/0/17	
^{MM}Dragonieri, 2012 [61]	The same as in de Genaro, 2010							
^{CRC}Altomare, 2013 [62]	IT	20/17	63±10	?	13/28	47±12	?	Training set
		7/8	67±11	?	6/4	56±10	?	Validation set

Table continued on following page

First author, year	Country	Cases			Controls			Comments
		M/F	Age	Smoking	M/F	Age	Smoking	
^{GC}Amal, 2013 [63]	LV	28/9	57±2	?	30/31	57±2	?	
^{GC}Xu Z, 2013 [64]	CN	28/9	58±9	15/?/?	23/9	51±14	14/?/?	Cn: gastric ulcer
					30/31	51±9	13/?/?	Cn: less severe conditions
					53/40	51^{WA}	27/?/?	Cn: all
^{HNC}Garcia, 2014 [65]	ES	10/1	61±11	?	6/4	45±15	5/0/5	
^{HNC}Gruber, 2014 [66]	IL	19/3	62±12	13/?/?	6/14	50±12	5/?/?	Cn: healthy
					14/7	55±14	12/?/?	Cn: benign conditions
^{HNC}Leunis, 2014 [67]	ND	25/11	59±?	36/0/0	10/13	48±?	23/0/0	
^{GC}Shehada, 2014 [68]	LV	19/11	60±10	9/0/19	24/53	55±16	14/0/63	
^{CRC}Wang C, 2014 [69]	CN	13/7	58±14	5/?/?	8/12	50±9	8/?/?	
^{OC}Amal, 2015 [70]	CN	0/48	51±11	0/0/48	0/48	48±9	0/0/48	Cn: healthy
					0/86	40±13	0/0/86	Cn: benign conditions
					0/134	43^{WA}	0/0/134	Cn: all
^{GC}Amal, 2015 [71]	LV	56/42	63±13	29/?/?	102/223	59±14	45/?/?	Cn: OLGIM 0-IV
					34/19	53±15	23/?/?	Cn: gastric ulcer
^{TC}Guo, 2015 [72]	CN	11/53	48^{WA}	13/?/?	6/26	40±13	4/?/?	
^{GC}Kumar, 2015 [73]	UK	64/17	62(53-71)	14/32/35	89/50	64(51-72)	24/32/73	

AT – Austria; AU – Australia; CA – Canada; CN – China; DE – Germany; ES – Spain; IL – Israel; IT – Italy; JP – Japan; LV – Latvia; ND – the Netherlands; PL – Poland; UK – United Kingdom; US – United States.

^{WA}weighted average. COPD - Chronic obstructive pulmonary disease; OLGIM - Operative Link on Gastric Intestinal Metaplasia Assessment.

References

1. Gordon SM, Szidon JP, Krotoszynski BK, Gibbons RD and O'Neill HJ. Volatile organic compounds in exhaled air from patients with lung cancer. Clinical chemistry. 1985; 31(8):1278-1282.
2. Preti G, Labows JN, Kostelc JG, Aldinger S and Daniele R. Analysis of lung air from patients with bronchogenic carcinoma and controls using gas chromatography-mass spectrometry. Journal of chromatography. 1988; 432:1-11.
3. Phillips M, Gleeson K, Hughes JM, Greenberg J, Cataneo RN, Baker L and McVay WP. Volatile organic compounds in breath as markers of lung cancer: a cross-sectional study. Lancet. 1999; 353(9168):1930-1933.
4. Di Natale C, Macagnano A, Martinelli E, Paolesse R, D'Arcangelo G, Roscioni C, Finazzi-Agro A and D'Amico A. Lung cancer identification by the analysis of breath by means of an array of non-selective gas sensors. Biosensors & bioelectronics. 2003; 18(10):1209-1218.
5. Phillips M, Cataneo RN, Cummin AR, Gagliardi AJ, Gleeson K, Greenberg J, Maxfield RA and Rom WN. Detection of lung cancer with volatile markers in the breath. Chest. 2003; 123(6):2115-2123.
6. Chen X, Cao MF, Li Y, Hu WJ, Wang P, Ying KJ and Pan HM. A study of an electronic nose for detection of lung cancer based on a virtual SAW gas sensors array and imaging recognition method. Measurement Science & Technology. 2005; 16(8):1535-1546.
7. Machado RF, Laskowski D, Deffenderfer O, Burch T, Zheng S, Mazzone PJ, Mekhail T, Jennings C, Stoller JK, Pyle J, Duncan J, Dweik RA and Erzurum SC. Detection of lung cancer by sensor array analyses of exhaled breath. American journal of respiratory and critical care medicine. 2005; 171(11):1286-1291.
8. Poli D, Carbognani P, Corradi M, Goldoni M, Acampa O, Balbi B, Bianchi L, Rusca M and Mutti A. Exhaled volatile organic compounds in patients with non-small cell lung cancer: cross sectional and nested short-term follow-up study. Respiratory research. 2005; 6:71.
9. Mazzone PJ, Hammel J, Dweik R, Na J, Czich C, Laskowski D and Mekhail T. Diagnosis of lung cancer by the analysis of exhaled breath with a colorimetric sensor array. Thorax. 2007; 62(7):565-568.
10. Phillips M, Altorki N, Austin JH, Cameron RB, Cataneo RN, Greenberg J, Kloss R, Maxfield RA, Munawar MI, Pass HI, Rashid A, Rom WN and Schmitt P. Prediction of lung cancer using volatile biomarkers in breath. Cancer biomarkers : section A of Disease markers. 2007; 3(2):95-109.
11. Steeghs MM, Cristescu SM, Munnik P, Zanen P and Harren FJ. An off-line breath sampling and analysis method suitable for large screening studies. Physiological measurement. 2007; 28(5):503-514.
12. Wehinger A, Schmid A, Mechtcheriakov S, Ledochowski M, Grabmer C, Gastl GA and Amann A. Lung cancer detection by proton transfer reaction mass-spectrometric analysis of human breath gas. International Journal of Mass Spectrometry. 2007; 265(1):49-59.
13. Phillips M, Altorki N, Austin JH, Cameron RB, Cataneo RN, Kloss R, Maxfield RA, Munawar MI, Pass HI, Rashid A, Rom WN, Schmitt P and Wai J. Detection of lung cancer using weighted digital analysis of breath biomarkers. Clinica chimica acta; international journal of clinical chemistry. 2008; 393(2):76-84.
14. Bajtarevic A, Ager C, Pienz M, Klieber M, Schwarz K, Ligor M, Ligor T, Filipiak W, Denz H, Fiegl M, Hilbe W, Weiss W, Lukas P, Jamnig H, Hackl M, Haidenberger A, et al. Noninvasive detection of lung cancer by analysis of exhaled breath. BMC cancer. 2009; 9:348.
15. Dragonieri S, Annema JT, Schot R, van der Schee MP, Spanevello A, Carratu P, Resta O, Rabe KF and Sterk PJ. An electronic nose in the discrimination of patients with non-small cell lung cancer and COPD. Lung cancer (Amsterdam, Netherlands). 2009; 64(2):166-170.
16. Ligor M, Ligor T, Bajtarevic A, Ager C, Pienz M, Klieber M, Denz H, Fiegl M, Hilbe W, Weiss W, Lukas P, Jamnig H, Hackl M, Buszewski B, Miekisch W, Schubert J, et al. Determination of volatile organic compounds in exhaled breath of patients with lung cancer using solid phase microextraction and gas chromatography mass spectrometry. Clinical chemistry and laboratory medicine : CCLM / FESCC. 2009; 47(5):550-560.
17. Peng G, Tisch U, Adams O, Hakim M, Shehada N, Broza YY, Billan S, Abdah-Bortnyak R, Kuten A and Haick H. Diagnosing lung cancer in exhaled breath using gold nanoparticles. Nature nanotechnology. 2009; 4(10):669-673.
18. Westhoff M, Litterst P, Freitag L, Urfer W, Bader S and Baumbach JI. Ion mobility spectrometry for the detection of volatile organic compounds in exhaled breath of patients with lung cancer: results of a pilot study. Thorax. 2009; 64(9):744-748.
19. D'Amico A, Pennazza G, Santonico M, Martinelli E, Roscioni C, Galluccio G, Paolesse R and Di Natale C. An investigation on electronic nose diagnosis of lung cancer. Lung cancer (Amsterdam, Netherlands). 2010; 68(2):170-176.
20. Fuchs P, Loeseken C, Schubert JK and Miekisch W. Breath gas aldehydes as biomarkers of lung cancer. International journal of cancer Journal international du cancer. 2010; 126(11):2663-2670.
21. Kischkel S, Miekisch W, Sawacki A, Straker EM, Trefz P, Amann A and Schubert JK. Breath biomarkers for lung cancer detection and assessment of smoking related effects - confounding variables, influence of normalization and statistical algorithms. Clinica Chimica Acta. 2010; 411(21-22):1637-1644.
22. Peng G, Hakim M, Broza YY, Billan S, Abdah-Bortnyak R, Kuten A, Tisch U and Haick H. Detection of lung, breast, colorectal, and prostate cancers from exhaled breath using a single array of nanosensors. Br J Cancer. 2010; 103(4):542-551.
23. Poli D, Goldoni M, Corradi M, Acampa O, Carbognani P, Internullo E, Casalini A and Mutti A. Determination of aldehydes in exhaled breath of patients with lung cancer by means of on-fiber-derivatisation SPME-GC/MS. Journal of chromatography B, Analytical technologies in the biomedical and life sciences. 2010; 878(27):2643-2651.
24. Song G, Qin T, Liu H, Xu GB, Pan YY, Xiong FX, Gu KS, Sun GP and Chen ZD. Quantitative breath analysis of volatile organic compounds of lung cancer patients. Lung cancer (Amsterdam, Netherlands). 2010; 67(2):227-231.
25. Hakim M, Billan S, Tisch U, Peng G, Dvrokind I, Marom O, Abdah-Bortnyak R, Kuten A and Haick H. Diagnosis of head-and-neck cancer from exhaled breath. British Journal of Cancer. 2011; 104(10):1649-1655.
26. Yu K, Wang Y, Yu J and Wang P. A Portable Electronic Nose Intended for Home Healthcare Based on a Mixed Sensor Array and Multiple Desorption Methods. Sensor Letters. 2011; 9(2):876-883.

27. Buszewski B, Ligor T, Jezierski T, Wenda-Piesik A, Walczak M and Rudnicka J. Identification of volatile lung cancer markers by gas chromatography-mass spectrometry: comparison with discrimination by canines. Analytical and bioanalytical chemistry. 2012; 404(1):141-146.
28. Hauschild AC, Baumbach JI and Baumbach J. Integrated statistical learning of metabolic ion mobility spectrometry profiles for pulmonary disease identification. Genetics and molecular research : GMR. 2012; 11(3):2733-2744.
29. Rudnicka J, Kowalkowski T, Ligor T and Buszewski B. Determination of volatile organic compounds as biomarkers of lung cancer by SPME-GC-TOF/MS and chemometrics. Journal of chromatography B, Analytical technologies in the biomedical and life sciences. 2011; 879(30):3360-3366.
30. Ulanowska A, Kowalkowski T, Trawinska E and Buszewski B. The application of statistical methods using VOCs to identify patients with lung cancer. Journal of breath research. 2011; 5(4):046008.
31. Mazzone PJ, Wang XF, Xu Y, Mekhail T, Beukemann MC, Na J, Kemling JW, Suslick KS and Sasidhar M. Exhaled breath analysis with a colorimetric sensor array for the identification and characterization of lung cancer. Journal of thoracic oncology : official publication of the International Association for the Study of Lung Cancer. 2012; 7(1):137-142.
32. Peled N, Hakim M, Bunn PA, Jr., Miller YE, Kennedy TC, Mattei J, Mitchell JD, Hirsch FR and Haick H. Non-invasive breath analysis of pulmonary nodules. Journal of thoracic oncology : official publication of the International Association for the Study of Lung Cancer. 2012; 7(10):1528-1533.
33. Santonico M, Lucantoni G, Pennazza G, Capuano R, Galluccio G, Roscioni C, La Delfa G, Consoli D, Martinelli E, Paolesse R, Di Natale C and D'Amico A. In situ detection of lung cancer volatile fingerprints using bronchoscopic air-sampling. Lung cancer (Amsterdam, Netherlands). 2012; 77(1):46-50.
34. Wang D, Yu K, Wang Y, Hu Y, Zhao C, Wang L, Ying K and Wang P. A Hybrid Electronic Noses' System Based on Mos-Saw Detection Units Intended for Lung Cancer Diagnosis. Journal of Innovative Optical Health Sciences. 2012; 5(1).
35. Wang Y, Hu Y, Wang D, Yu K, Wang L, Zou Y, Zhao C, Zhang X, Wang P and Ying K. The analysis of volatile organic compounds biomarkers for lung cancer in exhaled breath, tissues and cell lines. Cancer biomarkers : section A of Disease markers. 2012; 11(4):129-137.
36. Broza YY, Kremer R, Tisch U, Gevorkyan A, Shiban A, Best LA and Haick H. A nanomaterial-based breath test for short-term follow-up after lung tumor resection. Nanomedicine : nanotechnology, biology, and medicine. 2013; 9(1):15-21.
37. Bousamra M, 2nd, Schumer E, Li M, Knipp RJ, Nantz MH, van Berkel V and Fu XA. Quantitative analysis of exhaled carbonyl compounds distinguishes benign from malignant pulmonary disease. The Journal of thoracic and cardiovascular surgery. 2014; 148(3):1074-1080; discussion 1080-1071.
38. Filipiak W, Filipiak A, Sponring A, Schmid T, Zelger B, Ager C, Klodzinska E, Denz H, Pizzini A, Lucciarini P, Jamnig H, Troppmair J and Amann A. Comparative analyses of volatile organic compounds (VOCs) from patients, tumors and transformed cell lines for the validation of lung cancer-derived breath markers. Journal of breath research. 2014; 8(2):027111.
39. Fu XA, Li M, Knipp RJ, Nantz MH and Bousamra M. Noninvasive detection of lung cancer using exhaled breath. Cancer medicine. 2014; 3(1):174-181.
40. Handa H, Usuba A, Maddula S, Baumbach JI, Mineshita M and Miyazawa T. Exhaled breath analysis for lung cancer detection using ion mobility spectrometry. PloS one. 2014; 9(12):e114555.
41. Hubers AJ, Brinkman P, Boksem RJ, Rhodius RJ, Witte BI, Zwinderman AH, Heideman DAM, Duin S, Koning R, Steenbergen RDM, Snijders PJF, Smit EF, Sterk PJ and Thunnissen E. Combined sputum hypermethylation and eNose analysis for lung cancer diagnosis. Journal of Clinical Pathology. 2014; 67(8):707-711.
42. Ma H, Li X, Chen J, Wang H, Cheng T, Chen K and Xu S. Analysis of human breath samples of lung cancer patients and healthy controls with solid-phase microextraction (SPME) and flow-modulated comprehensive two-dimensional gas chromatography (GC x GC). Analytical Methods. 2014; 6(17):6841-6849.
43. Rudnicka J, Walczak M, Kowalkowski T, Jezierski T and Buszewski B. Determination of volatile organic compounds as potential markers of lung cancer by gas chromatography-mass spectrometry versus trained dogs. Sensors and Actuators B-Chemical. 2014; 202:615-621.
44. Xu H, Wei Y, Zhu L, Huang J, Li Y, Liu F, Wang S and Liu S. Bifunctional magnetic nanoparticles for analysis of aldehyde metabolites in exhaled breath of lung cancer patients. Journal of Chromatography A. 2014; 1324:29-35.
45. Zou Y, Zhang X, Chen X, Hu Y, Ying K and Wang P. Optimization of volatile markers of lung cancer to exclude interferences of non-malignant disease. Cancer biomarkers : section A of Disease markers. 2014; 14(5):371-379.
46. McWilliams A, Beigi P, Srinidhi A, Lam S and MacAulay C. Sex and Smoking Status Effects on the Early Detection of Early Lung Cancer in High-Risk Smokers using an Electronic Nose. IEEE transactions on bio-medical engineering. 2015.
47. Hietanen E, Bartsch H, Bereziat JC, Camus AM, McClinton S, Eremin O, Davidson L and Boyle P. Diet and oxidative stress in breast, colon and prostate cancer patients: a case-control study. European journal of clinical nutrition. 1994; 48(8):575-586.
48. Phillips M, Cataneo RN, Ditkoff BA, Fisher P, Greenberg J, Gunawardena R, Kwon CS, Rahbari-Oskoui F and Wong C. Volatile markers of breast cancer in the breath. The breast journal. 2003; 9(3):184-191.
49. Phillips M, Cataneo RN, Ditkoff BA, Fisher P, Greenberg J, Gunawardena R, Kwon CS, Tietje O and Wong C. Prediction of breast cancer using volatile biomarkers in the breath. Breast cancer research and treatment. 2006; 99(1):19-21.
50. Phillips M, Cataneo RN, Saunders C, Hope P, Schmitt P and Wai J. Volatile biomarkers in the breath of women with breast cancer. Journal of breath research. 2010; 4(2):026003.
51. Patterson SG, Bayer CW, Hendry RJ, Sellers N, Lee KS, Vidakovic B, Mizaikoff B and Gabram-Mendola SG. Breath analysis by mass spectrometry: a new tool for breast cancer detection? The American surgeon. 2011; 77(6):747-751.

52. Shuster G, Gallimidi Z, Reiss AH, Dovgolevsky E, Billan S, Abdah-Bortnyak R, Kuten A, Engel A, Shiban A, Tisch U and Haick H. Classification of breast cancer precursors through exhaled breath. Breast cancer research and treatment. 2011; 126(3):791-796.
53. Mangler M, Freitag C, Lanowska M, Staeck O, Schneider A and Speiser D. Volatile organic compounds (VOCs) in exhaled breath of patients with breast cancer in a clinical setting. Ginekologia polska. 2012; 83(10):730-736.
54. Li J, Peng Y, Liu Y, Li W, Jin Y, Tang Z and Duan Y. Investigation of potential breath biomarkers for the early diagnosis of breast cancer using gas chromatography-mass spectrometry. Clinica chimica acta; international journal of clinical chemistry. 2014; 436:59-67.
55. Phillips M, Beatty JD, Cataneo RN, Huston J, Kaplan PD, Lalisang RI, Lambin P, Lobbes MB, Mundada M, Pappas N and Patel U. Rapid point-of-care breath test for biomarkers of breast cancer and abnormal mammograms. PloS one. 2014; 9(3):e90226.
56. Wang C, Sun B, Guo L, Wang X, Ke C, Liu S, Zhao W, Luo S, Guo Z, Zhang Y, Xu G and Li E. Volatile organic metabolites identify patients with breast cancer, cyclomastopathy, and mammary gland fibroma. Scientific reports. 2014; 4:5383.
57. Rieder J, Lirk P, Ebenbichler C, Gruber G, Prazeller P, Lindinger W and Amann A. Analysis of volatile organic compounds: possible applications in metabolic disorders and cancer screening. Wiener klinische Wochenschrift. 2001; 113(5-6):181-185.
58. Qin T, Liu H, Song Q, Song G, Wang HZ, Pan YY, Xiong FX, Gu KS, Sun GP and Chen ZD. The screening of volatile markers for hepatocellular carcinoma. Cancer epidemiology, biomarkers & prevention : a publication of the American Association for Cancer Research, cosponsored by the American Society of Preventive Oncology. 2010; 19(9):2247-2253.
59. Chapman EA, Thomas PS, Stone E, Lewis C and Yates DH. A breath test for malignant mesothelioma using an electronic nose. The European respiratory journal. 2012; 40(2):448-454.
60. de Gennaro G, Dragonieri S, Longobardi F, Musti M, Stallone G, Trizio L and Tutino M. Chemical characterization of exhaled breath to differentiate between patients with malignant plueral mesothelioma from subjects with similar professional asbestos exposure. Analytical and bioanalytical chemistry. 2010; 398(7-8):3043-3050.
61. Dragonieri S, van der Schee MP, Massaro T, Schiavulli N, Brinkman P, Pinca A, Carratu P, Spanevello A, Resta O, Musti M and Sterk PJ. An electronic nose distinguishes exhaled breath of patients with Malignant Pleural Mesothelioma from controls. Lung cancer (Amsterdam, Netherlands). 2012; 75(3):326-331.
62. Altomare DF, Di Lena M, Porcelli F, Trizio L, Travaglio E, Tutino M, Dragonieri S, Memeo V and de Gennaro G. Exhaled volatile organic compounds identify patients with colorectal cancer. The British journal of surgery. 2013; 100(1):144-150.
63. Amal H, Leja M, Broza YY, Tisch U, Funka K, Liepniece-Karele I, Skapars R, Xu ZQ, Liu H and Haick H. Geographical variation in the exhaled volatile organic compounds. Journal of breath research. 2013; 7(4):047102.
64. Xu ZQ, Broza YY, Ionsecu R, Tisch U, Ding L, Liu H, Song Q, Pan YY, Xiong FX, Gu KS, Sun GP, Chen ZD, Leja M and Haick H. A nanomaterial-based breath test for distinguishing gastric cancer from benign gastric conditions. British journal of cancer. 2013; 108(4):941-950.
65. Garcia RA, Morales V, Martin S, Vilches E and Toledano A. Volatile Organic Compounds Analysis in Breath Air in Healthy Volunteers and Patients Suffering Epidermoid Laryngeal Carcinomas. Chromatographia. 2014; 77(5-6):501-509.
66. Gruber M, Tisch U, Jeries R, Amal H, Hakim M, Ronen O, Marshak T, Zimmerman D, Israel O, Amiga E, Doweck I and Haick H. Analysis of exhaled breath for diagnosing head and neck squamous cell carcinoma: a feasibility study. British journal of cancer. 2014; 111(4):790-798.
67. Leunis N, Boumans ML, Kremer B, Din S, Stobberingh E, Kessels AG and Kross KW. Application of an electronic nose in the diagnosis of head and neck cancer. The Laryngoscope. 2014; 124(6):1377-1381.
68. Shehada N, Bronstrup G, Funka K, Christiansen S, Leja M and Haick H. Ultrasensitive Silicon Nanowire for Real-World Gas Sensing: Noninvasive Diagnosis of Cancer from Breath Volatolome. Nano letters. 2015; 15(2):1288-1295.
69. Wang C, Ke C, Wang X, Chi C, Guo L, Luo S, Guo Z, Xu G, Zhang F and Li E. Noninvasive detection of colorectal cancer by analysis of exhaled breath. Analytical and bioanalytical chemistry. 2014; 406(19):4757-4763.
70. Amal H, Shi DY, Ionescu R, Zhang W, Hua QL, Pan YY, Tao L, Liu H and Haick H. Assessment of ovarian cancer conditions from exhaled breath. International journal of cancer Journal international du cancer. 2015; 136(6):E614-622.
71. Amal H, Leja M, Funka K, Skapars R, Sivins A, Ancans G, Liepniece-Karele I, Kikuste I, Lasina I and Haick H. Detection of precancerous gastric lesions and gastric cancer through exhaled breath. Gut. 2015.
72. Guo L, Wang C, Chi C, Wang X, Liu S, Zhao W, Ke C, Xu G and Li E. Exhaled breath volatile biomarker analysis for thyroid cancer. Translational research : the journal of laboratory and clinical medicine. 2015.
73. Kumar S, Huang J, Abbassi-Ghadi N, Mackenzie HA, Veselkov KA, Hoare JM, Lovat LB, Spanel P, Smith D and Hanna GB. Mass Spectrometric Analysis of Exhaled Breath for the Identification of Volatile Organic Compound Biomarkers in Esophageal and Gastric Adenocarcinoma. Annals of surgery. 2015.

Table S2. Description of breath collection procedure and patients recruitment: differences in proportions of age, sex and smoking distributions between cases and controls ("yes" if ≤10 % units), collected breath, collection time and restrictions, analysis time and inclusion criteria for participants in all studies.

ref		Difference in			Collected air	Restrictions	Quality assurance and (collection time)	Analysis time after collection	Exclusion/inclusion criteria	Comments
		sex	age	smoking						
[1]		yes	-	no	tidal	-	5min lung washout	-	Patients: no therapy; no comorbidities<br>Controls: no pulmonary/systematic disease	
[2]	Gr 1	no	yes	yes	alveolar	-	VOCs related to food and drugs were checked	≤1 week	Patients: no therapy<br>Gr 1: no chronic/acute pulmonary disease; no changes on chest roentgenogram; no industrial dust exposure; no drugs.<br>Gr 2 - no diseases, no drugs	Gr 1 - controls (age-matched)
	Gr 2	no	no	yes						Gr 2 - controls (younger)
[3]		yes	yes	no	alveolar	no food, drinks	room air analyzed; (morning)	At once	Patients: no therapy<br>Controls: no cancer	
[4]	Gr 1	-	-	-	vital capacity	no food	(morning)	At once	Patients: no therapy<br>Gr 1: no apparent disease, no drugs	Gr 1 - controls
	Gr 2	-	-	-						Gr 2 - cases after surgery
[5]	Gr 1	no	yes	no	alveolar	-	room air analyzed	-	Patients: no therapy<br>Gr 1: no cancer, chronic disease	Gr 1 - healthy
	Gr 2	no	yes	-						Gr 2 - abnormal chest x-rays
[6]	Set 1	-	-	-	-	2h no food	-	At once	Patients: under different treatment regimes<br>Controls: 7 had bronchitis	Set 1 - training
	Set 2	-	-	-						Set 2 - validation
[7]	Set 1	no	no	yes	vital capacity, no nasal	-	-	At once	Set 1: healthy: no pulmonary symptoms, history of pulmonary disease, abnormal lung function<br>Set 2: Patients: no therapy<br>all: no acute disease exacerbation, history of cancer, diabetes, immunosuppression, coronary artery disease	Set 1 - training
	Set 2	no	no	-						Set 2 - validation
[8]	Gr 1	no	no	yes	alveolar	rest 1h; Gr 2 - 1h no smoking	room air analyzed	~30 min	Patients: no therapy<br>Gr 1: no pulmonary symptoms, history of pulmonary disease, abnormal lung spirometry results<br>controls: no chronic bronchitis	Gr 1 - healthy non-smokers
	Gr 2	yes	no	no						Gr 2 - healthy smokers
	Gr 3	yes	yes	yes						Gr 3 - COPD
[9]		yes	yes	no	tidal	-	room air analyzed	At once	Patients: different treatment regimes<br>Controls: no lung conditions, cardiopulmonary symptoms	
[10, 11]		yes	yes	no	alveolar	-	room air analyzed	-	Patients: no therapy<br>Controls: no cancer	
[12]		-	yes	yes	alveolar	no restrictions	room air analyzed	~54 h	Patients: no therapy	
[13]		no	no	no	tidal	-	room air analyzed	≤12 h[1]	Patients: no therapy	
[14, 15]		no	no	no	mixed alveolar	rest 5min; 1h no food	room air analyzed; (any time)	3-6 h	Patients: different treatment regimes	
[16]	Gr 1	no	yes	no	vital capacity	2h no food, drinks, no smoking that day	5min lung washout; room air analyzed	At once	Patients: no therapy, no upper/lower respiratory tract infections (in 4 weeks), no systematic disease/other cancer<br>Gr 1: no diseases<br>Gr 2: no asthma, pulmonary/cardiovascular diseases	Gr 1 - healthy
	Gr 2	no	yes	no						Gr 2 - COPD
[17]		-	-	no	alveolar	1h no coffee, 12h no alcohol	5min lung washout	≤2 days	Patients: no therapy<br>controls: no restrictions, 3 had asthma, 2-asthma+sinusitus	

Table continued on following page

Continued

ref		Difference in sex	Difference in age	Difference in smoking	Collected air	Restrictions	Quality assurance and (collection time)	Analysis time after collection	Exclusion/inclusion criteria	Comments
[18]		yes	no	yes	alveolar	-	room air analyzed	At once	Patients: no therapy<br>Controls: no cancer, no disease	
[19]	Gr 1	-	-	yes	alveolar	no food, drinks	(morning)	At once	Patients: no current therapy<br>Gr 1: no other disease<br>Gr 2: 16 had COPD, 5-bronchitis, 7-other diseases	Gr 1 - healthy
	Gr 2	-	yes	yes						Gr 2 - other diseases
[20]		no	no	no	alveolar	rest 10min	room air analyzed	≤6 h	Patients: no current therapy<br>Controls: no cancer, no chronic disease	
[21]	Gr 1	no	no	no	alveolar	rest 10min; 1h no food, drinks	room air analyzed	≤6 h	Patients: no therapy<br>Gr 1, Gr 2: no COPD	Gr 1 - healthy smokers
	Gr 2	no	no	yes						Gr 2 - healthy non-smokers
[22]		-	-	no[2]	alveolar	12h no coffee, alcohol	3-5min lung washout (morning)	≤4 days	Patients: no therapy<br>Controls: no restrictions	
[23]	Gr 1	no	no	no	alveolar	-	-	≤2 h	Patients: no therapy<br>Controls: no pulmonary disease, no previous cancer	Gr 1=Gr 2+Gr 3
	Gr 2	-	-	no						Gr 2 - cases smokers
	Gr 3	-	-	no						Gr 3 - cases non- and ex-smokers
[24]		yes	no	yes	vital capacity	no food, drinks	room air analyzed; (morning)	≤6 h	Patients: no current therapy	
[25]		-	-	-	alveolar	-	lung washout, room air analyzed	≤3 days	Patients: no therapy	
[26]		-	-	no	alveolar	no food	stay 10min in ventilated room; (morning)	At once	not described	
[27]		-	-	-	alveolar	no restrictions	room air analyzed	-	not described	
[28]	Gr 1	-	-	-	alveolar	-	-	At once	-	Gr 1 - healthy
	Gr 2	-	-	-						Gr 2 - COPD
[29]		no	-	no	-	-	room air analyzed	-	-	
[30]		no	-	yes	alveolar	-	room air analyzed	At once	Controls: no restrictions, questionnaire filled	
[31]		yes	yes	yes	tidal	no restrictions	room air analyzed	At once	Patients: no therapy<br>Controls: no cancer in 5 years, not requiring continuous supplemental oxygen, no under long-term immunosuppressive therapies. All had COPD or family lung cancer history	
[32]		no	yes	yes	alveolar	-	3min lung washout	-	Patients: no therapy<br>Controls: some had heart disease or COPD	
[33]		yes	yes	yes	alveolar	-		At once	Patients: under different treatment regimes<br>Controls: benign conditions; 2 were under therapy	
[34]		no	-	no	-	12h no food, smoking; no high-fat dinner the night before	gargle water before breath sampling; (morning)	At once	Patients: no therapy<br>Controls: no respiratory symptoms, no acute illnesses/comorbidities (diabetes, bronchitis, peptic ulcer, oxyhepatitis and coronary heart disease)	

Table continued on following page

Continued

ref		Difference in sex	Difference in age	Difference in smoking	Collected air	Restrictions	Quality assurance and (collection time)	Analysis time after collection	Exclusion/inclusion criteria	Comments
[35]	Gr 1	no	no	no	alveolar	12h no food, smoking	stay 30min in ventilated room; room air analyzed; (morning)	-	Patients: no therapy	Gr 1 - healthy
	Gr 2	yes	yes	no						Gr 2 - benign
	Gr 3	no	no	no						Gr 3=Gr 1+Gr 2
[36]		yes	yes	yes	alveolar	1h no food, coffee, smoking	3min lung washout	≤6 h	Patients: no therapy<br>Controls: hospitalized for benign conditions	
[37]	Gr 1	-	no	no	vital capacity	-	(morning)	At once	Patients: no therapy<br>Gr 1: no active pulmonary disease	Gr 1 - healthy
	Gr 2	-	no	no						Gr 2 - benign
[38]		no	no	yes	alveolar	rest 10min; 2h no food	room air analyzed; (morning)	≤5 h	-	
[39]		-	-	-	tidal+ alveolar	-	-	At once	Patients: no therapy	
[40]		yes	no	no	tidal, no dead space	-	-	At once	Patients: no therapy	
[41]	Set 1	no	yes	yes	vital capacity	2h no food, drinks, smoking	5min lung washout, room air analyzed	At once	Patients: no therapy<br>Controls: no cancer, majority had COPD	Set 1 - training
	Set 2	yes	no	yes						Set 2 - validation
[42]		no	no	yes	alveolar	30min no food, drinks, 2h no brushing, 10h no smoking, alcohol, 24h no spicy food	Stay 30min in ventilated room; room air analyzed (before lunch)	≤24 h	Patients: under different treatment regimes<br>Controls: no history of severe COPD, asthma, neurological disorder or Wilson's disease, diabetes, no sedatives or narcotics within 48 h	
[43]		no	yes	-	alveolar	-	room air analyzed	3-4 h	-	
[44]		-	-	-	tidal	-	-	-	not described	
[45]	Set 1	yes	yes	yes	alveolar	12h no food	rinse out mouth with distilled water; room air analyzed; (morning)	At once	Patients: no therapy	Set 1: training
	Set 2	-	-	-						Set 2: validation
[46]		yes	yes	no	vital capacity	-	room air analyzed	At once	Patients: no therapy (1 patient had therapy for breast cancer); 4 patients had previous cancer diagnosis, comorbidities present<br>Controls: "High risk smokers"; comorbidities present	
[47]		yes	yes[3]	yes3	alveolar	-	room air analyzed		Patients: 5 had comorbidities<br>Controls: hospitalized for benign conditions	
[48, 49]	Gr 1	yes	yes	no	alveolar	no food, drinks	room air analyzed; (morning)	-	Patients: no therapy<br>Gr 1: no cancer, no chronic disease<br>Gr 2: no cancer	Gr 1 - healthy
	Gr 2	yes	yes	-						Gr 2 - abnormal mammogram
[50]		yes	yes	yes	alveolar	no restrictions	room air analyzed	-	Patients: no therapy<br>Controls: no cancer	

Table continued on following page

Continued

ref		Difference in sex	Difference in age	Difference in smoking	Collected air	Restrictions	Quality assurance and (collection time)	Analysis time after collection	Exclusion/inclusion criteria	Comments
[51]		yes	yes	-	alveolar	2h no food, drinks	VOCs related to food, smoking, cosmetics were excluded	-	Patients: no therapy	
[52]	Gr 1	yes	-	yes	alveolar	-	-	-	Patients: no therapy<br>Controls: no exclusions, all volunteers	Gr 1 - healthy
	Gr 2	yes	-	yes						Gr 2 - precursors
[53]		yes	yes	-	alveolar	8h no food, drinks, tooth brushing	room air analyzed; (morning)	-	Patients: no therapy<br>Controls: no diseases	
[54]	Gr 1	yes	-	yes	alveolar	rest 10min	room air analyzed	≤12 h	Patients: no therapy<br>Gr 1: no cancer, no breast disease	Gr 1 - healthy
	Gr 2	yes	-	yes						Gr 2 - benign
[55]		yes	-	-	alveolar	-	room air analyzed	At once	Patients: no therapy<br>Controls: no cancer, had palpable breast mass	
[56]	Gr 1	yes	yes	yes	alveolar	no food, drinks	room air analyzed; (morning)	≤3 h	Patients: no therapy<br>Gr1: no currently breast feeding or pregnant, no congenital disease, cancer, COPD, asthma, tuberculosis, other pulmonary diseases; chronic inflammatory or infectious disease; no manifestation of any acute disease symptoms during 2 weeks	Gr 1 - healthy
	Gr 2	yes	no	yes						Gr 2 - cyclomastopathy
	Gr 3	yes	no	yes						Gr 3 - mammary gland fibroma
[57]		-	-	-	vital capacity	rest 15min; 2h no food, smoking, gum chewing	room air analyzed; (morning)	-	Controls: few had Systemic Inflammatory Response Syndrome	
[58]		no	yes	yes	vital capacity	no food, drinks	room air analyzed; (morning)	At once	Patients: no therapy<br>Controls: no chronic diseases	
[59]	Gr 1	yes	yes	yes	vital capacity	20min rest; 1.5h no food, drinks	mouth wash with distillate water	At once	Gr 1: no recent respiratory tract infection, an acute exacerbation of any underlying respiratory disease in the past 4 weeks and/or other current uncontrolled medical conditions	Gr 1 - healthy
	Gr 2	yes	yes	yes						Gr 2 - other diseases
[60, 61]	Gr 1	no	yes	yes	vital capacity	3h no food, drinks	5min lung washout; room air analyzed	At once	Patients: no current treatment, no other pulmonary or cardiovascular abnormalities.<br>Gr 1: no history of upper or lower respiratory tract infection during the past 4 weeks, no asthma, COPD, systemic diseases (such as diabetes), no cancer, no drugs	Gr 1 - healthy
	Gr 2	no	yes	yes						Gr 2 - exposed to asbestos
[62]	Set 1	no	no	-	vital capacity	rest 10min; 3h no food, drinks	5min lung washout	At once	Patients: no therapy; no asthma, severe COPD, unstable diabetes, another malignancy.<br>Controls: no inflammatory bowel disease or diverticulitis	Set 1 - training
	Set 2	no	no	-						Set 2 - validation
[63]		no	yes	-	alveolar	no food, drinks; 2h no smoking, alcohol	3min lung washout; room air analyzed; (morning)	≤3 months	Controls: no grade III–IV atrophic gastritis, no patients following stomach resections	

Table continued on following page

Continued

ref		Difference in			Collected air	Restrictions	Quality assurance and (collection time)	Analysis time after collection	Exclusion/inclusion criteria	Comments
		sex	age	smoking						
[64]	Gr 1	yes	yes	yes	alveolar	1h rest, 24h no heavy physical activity; 12h no food, smoking, alcohol	lung washout; room air analyzed; (morning)	≤4 months	Patients: no therapy<br>Gr1 and Gr2: no medication affecting gastric acid secretion and/or antibiotics in 1 month before the testing	Gr 1 - gastric ulcer
	Gr 2	no	yes	no						Gr 2 - less severe conditions
	Gr 3	no	yes	no						Gr 3=Gr 1+Gr 2
[65]		no	no	-	-	8h no food, drinks	room air analyzed; (morning)	≤12 h[1](if possible)	-	
[66]	Gr 1	no	no	no	alveolar	12h no food, drinks, alcohol	3-5min lung washout, room air analyzed; (morning)	-	Patients: no therapy<br>Gr 1: no previous cancer, active infectious disease, present antibiotic treatment, pregnancy or lactation	Gr 1 - healthy
	Gr 2	no	yes	no						Gr 2 - benign
[67]		no	no	yes	whole[4]	8h no food	(morning)	At once	Patients: no therapy<br>Controls: visiting hospital with some conditions	
[68]		no	yes	no	alveolar	2h no food, smoking, alcohol	3min lung washout; (morning)	≤3 months	Controls: no exclusions; some had gastric ulcer or other gastric disease	
[69]		no	yes	no	alveolar	8h no food, drinks	room air analyzed; (morning)	≤3 h	Patients: no therapy; no pregnancy, lactation; congenital disease; family history of mental illness; no current chronic inflammatory disease; symptoms of an acute disease during the 2 weeks; no a history of infectious disease.<br>Controls: no history of malignancies or infectious disease.	
[70]	Gr 1	yes	no	yes	alveolar	2h no food, coffee, alcohol; no cosmetics	3min lung washout; room air analyzed	≤4 months	G2: no cancer, no chronic disease, no autoimmune diseases	Gr 1 - benign
	Gr 2	yes	yes	yes						Gr 2 - healthy
	Gr 3	yes	yes	yes						Gr 3=Gr 1+Gr 2
[71]	Gr 1	no	yes	yes	alveolar	no food, drinks; 3h no smoking	3min lung washout; room air analyzed; (morning)	≤6 months	Patients: no therapy<br>Gr 1: no past stomach surgery<br>Gr 2: no dysplasia, no past stomach surgery	Gr 1 - OLGIM 0-IV
	Gr 2	yes	no	no						Gr 2 - peptic ulcer
[72]		yes	yes	yes	alveolar	no food, drinks	room air analyzed; (morning)	≤3 h	All: no pregnancy, lactation, no congenital, chronic inflammatory, acute (in 2 weeks) or infectious disease, no family history of mental illness	
[73]		no	yes	yes	mixed alveolar	6h no food, drinks	room air analyzed	≤1 h	Patients: no therapy; no squamous cell carcinoma of the upper gastrointestinal tract<br>All: no liver disease, small bowel/colonic pathology, other cancers, no acute infection. Some had comorbidities<br>Controls: 16 had Barrett's metaplasia, 62-benign condition	

[1]analysis on the same day; [2]lung cancer cases; [3]authors stated in a text that cases and controls were age- and smoking status-matched. [4]multiple repeated exhalation; COPD-Chronic obstructive pulmonary disease; OLGIM - Operative Link on Gastric Intestinal Metaplasia Assessment.

References

1. Gordon SM, Szidon JP, Krotoszynski BK, Gibbons RD and O'Neill HJ. Volatile organic compounds in exhaled air from patients with lung cancer. Clinical chemistry. 1985; 31(8):1278-1282.
2. Preti G, Labows JN, Kostelc JG, Aldinger S and Daniele R. Analysis of lung air from patients with bronchogenic carcinoma and controls using gas chromatography-mass spectrometry. Journal of chromatography. 1988; 432:1-11.
3. Phillips M, Gleeson K, Hughes JM, Greenberg J, Cataneo RN, Baker L and McVay WP. Volatile organic compounds in breath as markers of lung cancer: a cross-sectional study. Lancet. 1999; 353(9168):1930-1933.
4. Di Natale C, Macagnano A, Martinelli E, Paolesse R, D'Arcangelo G, Roscioni C, Finazzi-Agro A and D'Amico A. Lung cancer identification by the analysis of breath by means of an array of non-selective gas sensors. Biosensors & bioelectronics. 2003; 18(10):1209-1218.
5. Phillips M, Cataneo RN, Cummin AR, Gagliardi AJ, Gleeson K, Greenberg J, Maxfield RA and Rom WN. Detection of lung cancer with volatile markers in the breath. Chest. 2003; 123(6):2115-2123.
6. Chen X, Cao MF, Li Y, Hu WJ, Wang P, Ying KJ and Pan HM. A study of an electronic nose for detection of lung cancer based on a virtual SAW gas sensors array and imaging recognition method. Measurement Science & Technology. 2005; 16(8):1535-1546.
7. Machado RF, Laskowski D, Deffenderfer O, Burch T, Zheng S, Mazzone PJ, Mekhail T, Jennings C, Stoller JK, Pyle J, Duncan J, Dweik RA and Erzurum SC. Detection of lung cancer by sensor array analyses of exhaled breath. American journal of respiratory and critical care medicine. 2005; 171(11):1286-1291.
8. Poli D, Carbognani P, Corradi M, Goldoni M, Acampa O, Balbi B, Bianchi L, Rusca M and Mutti A. Exhaled volatile organic compounds in patients with non-small cell lung cancer: cross sectional and nested short-term follow-up study. Respiratory research. 2005; 6:71.
9. Mazzone PJ, Hammel J, Dweik R, Na J, Czich C, Laskowski D and Mekhail T. Diagnosis of lung cancer by the analysis of exhaled breath with a colorimetric sensor array. Thorax. 2007; 62(7):565-568.
10. Phillips M, Altorki N, Austin JH, Cameron RB, Cataneo RN, Greenberg J, Kloss R, Maxfield RA, Munawar MI, Pass HI, Rashid A, Rom WN and Schmitt P. Prediction of lung cancer using volatile biomarkers in breath. Cancer biomarkers : section A of Disease markers. 2007; 3(2):95-109.
11. Phillips M, Altorki N, Austin JH, Cameron RB, Cataneo RN, Kloss R, Maxfield RA, Munawar MI, Pass HI, Rashid A, Rom WN, Schmitt P and Wai J. Detection of lung cancer using weighted digital analysis of breath biomarkers. Clinica chimica acta; international journal of clinical chemistry. 2008; 393(2):76-84.
12. Steeghs MM, Cristescu SM, Munnik P, Zanen P and Harren FJ. An off-line breath sampling and analysis method suitable for large screening studies. Physiological measurement. 2007; 28(5):503-514.
13. Wehinger A, Schmid A, Mechtcheriakov S, Ledochowski M, Grabmer C, Gastl GA and Amann A. Lung cancer detection by proton transfer reaction mass-spectrometric analysis of human breath gas. International Journal of Mass Spectrometry. 2007; 265(1):49-59.
14. Bajtarevic A, Ager C, Pienz M, Klieber M, Schwarz K, Ligor M, Ligor T, Filipiak W, Denz H, Fiegl M, Hilbe W, Weiss W, Lukas P, Jamnig H, Hackl M, Haidenberger A, et al. Noninvasive detection of lung cancer by analysis of exhaled breath. BMC cancer. 2009; 9:348.
15. Ligor M, Ligor T, Bajtarevic A, Ager C, Pienz M, Klieber M, Denz H, Fiegl M, Hilbe W, Weiss W, Lukas P, Jamnig H, Hackl M, Buszewski B, Miekisch W, Schubert J, et al. Determination of volatile organic compounds in exhaled breath of patients with lung cancer using solid phase microextraction and gas chromatography mass spectrometry. Clinical chemistry and laboratory medicine : CCLM / FESCC. 2009; 47(5):550-560.
16. Dragonieri S, Annema JT, Schot R, van der Schee MP, Spanevello A, Carratu P, Resta O, Rabe KF and Sterk PJ. An electronic nose in the discrimination of patients with non-small cell lung cancer and COPD. Lung cancer (Amsterdam, Netherlands). 2009; 64(2):166-170.
17. Peng G, Tisch U, Adams O, Hakim M, Shehada N, Broza YY, Billan S, Abdah-Bortnyak R, Kuten A and Haick H. Diagnosing lung cancer in exhaled breath using gold nanoparticles. Nature nanotechnology. 2009; 4(10):669-673.
18. Westhoff M, Litterst P, Freitag L, Urfer W, Bader S and Baumbach JI. Ion mobility spectrometry for the detection of volatile organic compounds in exhaled breath of patients with lung cancer: results of a pilot study. Thorax. 2009; 64(9):744-748.
19. D'Amico A, Pennazza G, Santonico M, Martinelli E, Roscioni C, Galluccio G, Paolesse R and Di Natale C. An investigation on electronic nose diagnosis of lung cancer. Lung cancer (Amsterdam, Netherlands). 2010; 68(2):170-176.
20. Fuchs P, Loeseken C, Schubert JK and Miekisch W. Breath gas aldehydes as biomarkers of lung cancer. International journal of cancer Journal international du cancer. 2010; 126(11):2663-2670.
21. Kischkel S, Miekisch W, Sawacki A, Straker EM, Trefz P, Amann A and Schubert JK. Breath biomarkers for lung cancer detection and assessment of smoking related effects - confounding variables, influence of normalization and statistical algorithms. Clinica Chimica Acta. 2010; 411(21-22):1637-1644.
22. Peng G, Hakim M, Broza YY, Billan S, Abdah-Bortnyak R, Kuten A, Tisch U and Haick H. Detection of lung, breast, colorectal, and prostate cancers from exhaled breath using a single array of nanosensors. British journal of cancer. 2010; 103(4):542-551.
23. Poli D, Goldoni M, Corradi M, Acampa O, Carbognani P, Internullo E, Casalini A and Mutti A. Determination of aldehydes in exhaled breath of patients with lung cancer by means of on-fiber-derivatisation SPME-GC/MS. Journal of chromatography B, Analytical technologies in the biomedical and life sciences. 2010; 878(27):2643-2651.
24. Song G, Qin T, Liu H, Xu GB, Pan YY, Xiong FX, Gu KS, Sun GP and Chen ZD. Quantitative breath analysis of volatile organic compounds of lung cancer patients. Lung cancer (Amsterdam, Netherlands). 2010; 67(2):227-231.
25. Hakim M, Billan S, Tisch U, Peng G, Dvrokind I, Marom O, Abdah-Bortnyak R, Kuten A and Haick H. Diagnosis of head-and-neck cancer from exhaled breath. British journal of cancer. 2011; 104(10):1649-1655.
26. Yu K, Wang Y, Yu J and Wang P. A Portable Electronic Nose Intended for Home Healthcare Based on a Mixed Sensor Array and Multiple Desorption Methods. Sensor Letters. 2011; 9(2):876-883.

27. Buszewski B, Ligor T, Jezierski T, Wenda-Piesik A, Walczak M and Rudnicka J. Identification of volatile lung cancer markers by gas chromatography-mass spectrometry: comparison with discrimination by canines. Analytical and bioanalytical chemistry. 2012; 404(1):141-146.
28. Hauschild AC, Baumbach JI and Baumbach J. Integrated statistical learning of metabolic ion mobility spectrometry profiles for pulmonary disease identification. Genetics and molecular research : GMR. 2012; 11(3):2733-2744.
29. Rudnicka J, Kowalkowski T, Ligor T and Buszewski B. Determination of volatile organic compounds as biomarkers of lung cancer by SPME-GC-TOF/MS and chemometrics. Journal of chromatography B, Analytical technologies in the biomedical and life sciences. 2011; 879(30):3360-3366.
30. Ulanowska A, Kowalkowski T, Trawinska E and Buszewski B. The application of statistical methods using VOCs to identify patients with lung cancer. Journal of breath research. 2011; 5(4):046008.
31. Mazzone PJ, Wang XF, Xu Y, Mekhail T, Beukemann MC, Na J, Kemling JW, Suslick KS and Sasidhar M. Exhaled breath analysis with a colorimetric sensor array for the identification and characterization of lung cancer. Journal of thoracic oncology : official publication of the International Association for the Study of Lung Cancer. 2012; 7(1):137-142.
32. Peled N, Hakim M, Bunn PA, Jr., Miller YE, Kennedy TC, Mattei J, Mitchell JD, Hirsch FR and Haick H. Non-invasive breath analysis of pulmonary nodules. Journal of thoracic oncology : official publication of the International Association for the Study of Lung Cancer. 2012; 7(10):1528-1533.
33. Santonico M, Lucantoni G, Pennazza G, Capuano R, Galluccio G, Roscioni C, La Delfa G, Consoli D, Martinelli E, Paolesse R, Di Natale C and D'Amico A. In situ detection of lung cancer volatile fingerprints using bronchoscopic air-sampling. Lung cancer (Amsterdam, Netherlands). 2012; 77(1):46-50.
34. Wang D, Yu K, Wang Y, Hu Y, Zhao C, Wang L, Ying K and Wang P. A Hybrid Electronic Noses' System Based on Mos-Saw Detection Units Intended for Lung Cancer Diagnosis. Journal of Innovative Optical Health Sciences. 2012; 5(1).
35. Wang Y, Hu Y, Wang D, Yu K, Wang L, Zou Y, Zhao C, Zhang X, Wang P and Ying K. The analysis of volatile organic compounds biomarkers for lung cancer in exhaled breath, tissues and cell lines. Cancer biomarkers : section A of Disease markers. 2012; 11(4):129-137.
36. Broza YY, Kremer R, Tisch U, Gevorkyan A, Shiban A, Best LA and Haick H. A nanomaterial-based breath test for short-term follow-up after lung tumor resection. Nanomedicine : nanotechnology, biology, and medicine. 2013; 9(1):15-21.
37. Bousamra M, 2nd, Schumer E, Li M, Knipp RJ, Nantz MH, van Berkel V and Fu XA. Quantitative analysis of exhaled carbonyl compounds distinguishes benign from malignant pulmonary disease. The Journal of thoracic and cardiovascular surgery. 2014; 148(3):1074-1080; discussion 1080-1071.
38. Filipiak W, Filipiak A, Sponring A, Schmid T, Zelger B, Ager C, Klodzinska E, Denz H, Pizzini A, Lucciarini P, Jamnig H, Troppmair J and Amann A. Comparative analyses of volatile organic compounds (VOCs) from patients, tumors and transformed cell lines for the validation of lung cancer-derived breath markers. Journal of breath research. 2014; 8(2):027111.
39. Fu XA, Li M, Knipp RJ, Nantz MH and Bousamra M. Noninvasive detection of lung cancer using exhaled breath. Cancer medicine. 2014; 3(1):174-181.
40. Handa H, Usuba A, Maddula S, Baumbach JI, Mineshita M and Miyazawa T. Exhaled breath analysis for lung cancer detection using ion mobility spectrometry. PloS one. 2014; 9(12):e114555.
41. Hubers AJ, Brinkman P, Boksem RJ, Rhodius RJ, Witte BI, Zwinderman AH, Heideman DAM, Duin S, Koning R, Steenbergen RDM, Snijders PJF, Smit EF, Sterk PJ and Thunnissen E. Combined sputum hypermethylation and eNose analysis for lung cancer diagnosis. Journal of Clinical Pathology. 2014; 67(8):707-711.
42. Ma H, Li X, Chen J, Wang H, Cheng T, Chen K and Xu S. Analysis of human breath samples of lung cancer patients and healthy controls with solid-phase microextraction (SPME) and flow-modulated comprehensive two-dimensional gas chromatography (GC x GC). Analytical Methods. 2014; 6(17):6841-6849.
43. Rudnicka J, Walczak M, Kowalkowski T, Jezierski T and Buszewski B. Determination of volatile organic compounds as potential markers of lung cancer by gas chromatography-mass spectrometry versus trained dogs. Sensors and Actuators B-Chemical. 2014; 202:615-621.
44. Xu H, Wei Y, Zhu L, Huang J, Li Y, Liu F, Wang S and Liu S. Bifunctional magnetic nanoparticles for analysis of aldehyde metabolites in exhaled breath of lung cancer patients. Journal of Chromatography A. 2014; 1324:29-35.
45. Zou Y, Zhang X, Chen X, Hu Y, Ying K and Wang P. Optimization of volatile markers of lung cancer to exclude interferences of non-malignant disease. Cancer biomarkers : section A of Disease markers. 2014; 14(5):371-379.
46. McWilliams A, Beigi P, Srinidhi A, Lam S and MacAulay C. Sex and Smoking Status Effects on the Early Detection of Early Lung Cancer in High-Risk Smokers using an Electronic Nose. IEEE transactions on bio-medical engineering. 2015.
47. Hietanen E, Bartsch H, Bereziat JC, Camus AM, McClinton S, Eremin O, Davidson L and Boyle P. Diet and oxidative stress in breast, colon and prostate cancer patients: a case-control study. European journal of clinical nutrition. 1994; 48(8):575-586.
48. Phillips M, Cataneo RN, Ditkoff BA, Fisher P, Greenberg J, Gunawardena R, Kwon CS, Rahbari-Oskoui F and Wong C. Volatile markers of breast cancer in the breath. The breast journal. 2003; 9(3):184-191.
49. Phillips M, Cataneo RN, Ditkoff BA, Fisher P, Greenberg J, Gunawardena R, Kwon CS, Tietje O and Wong C. Prediction of breast cancer using volatile biomarkers in the breath. Breast cancer research and treatment. 2006; 99(1):19-21.
50. Phillips M, Cataneo RN, Saunders C, Hope P, Schmitt P and Wai J. Volatile biomarkers in the breath of women with breast cancer. Journal of breath research. 2010; 4(2):026003.
51. Patterson SG, Bayer CW, Hendry RJ, Sellers N, Lee KS, Vidakovic B, Mizaikoff B and Gabram-Mendola SG. Breath analysis by mass spectrometry: a new tool for breast cancer detection? The American surgeon. 2011; 77(6):747-751.

52. Shuster G, Gallimidi Z, Reiss AH, Dovgolevsky E, Billan S, Abdah-Bortnyak R, Kuten A, Engel A, Shiban A, Tisch U and Haick H. Classification of breast cancer precursors through exhaled breath. Breast cancer research and treatment. 2011; 126(3):791-796.
53. Mangler M, Freitag C, Lanowska M, Staeck O, Schneider A and Speiser D. Volatile organic compounds (VOCs) in exhaled breath of patients with breast cancer in a clinical setting. Ginekologia polska. 2012; 83(10):730-736.
54. Li J, Peng Y, Liu Y, Li W, Jin Y, Tang Z and Duan Y. Investigation of potential breath biomarkers for the early diagnosis of breast cancer using gas chromatography-mass spectrometry. Clinica chimica acta; international journal of clinical chemistry. 2014; 436:59-67.
55. Phillips M, Beatty JD, Cataneo RN, Huston J, Kaplan PD, Lalisang RI, Lambin P, Lobbes MB, Mundada M, Pappas N and Patel U. Rapid point-of-care breath test for biomarkers of breast cancer and abnormal mammograms. PloS one. 2014; 9(3):e90226.
56. Wang C, Sun B, Guo L, Wang X, Ke C, Liu S, Zhao W, Luo S, Guo Z, Zhang Y, Xu G and Li E. Volatile organic metabolites identify patients with breast cancer, cyclomastopathy, and mammary gland fibroma. Scientific reports. 2014; 4:5383.
57. Rieder J, Lirk P, Ebenbichler C, Gruber G, Prazeller P, Lindinger W and Amann A. Analysis of volatile organic compounds: possible applications in metabolic disorders and cancer screening. Wiener klinische Wochenschrift. 2001; 113(5-6):181-185.
58. Qin T, Liu H, Song Q, Song G, Wang HZ, Pan YY, Xiong FX, Gu KS, Sun GP and Chen ZD. The screening of volatile markers for hepatocellular carcinoma. Cancer epidemiology, biomarkers & prevention : a publication of the American Association for Cancer Research, cosponsored by the American Society of Preventive Oncology. 2010; 19(9):2247-2253.
59. Chapman EA, Thomas PS, Stone E, Lewis C and Yates DH. A breath test for malignant mesothelioma using an electronic nose. The European respiratory journal. 2012; 40(2):448-454.
60. de Gennaro G, Dragonieri S, Longobardi F, Musti M, Stallone G, Trizio L and Tutino M. Chemical characterization of exhaled breath to differentiate between patients with malignant plueral mesothelioma from subjects with similar professional asbestos exposure. Analytical and bioanalytical chemistry. 2010; 398(7-8):3043-3050.
61. Dragonieri S, van der Schee MP, Massaro T, Schiavulli N, Brinkman P, Pinca A, Carratu P, Spanevello A, Resta O, Musti M and Sterk PJ. An electronic nose distinguishes exhaled breath of patients with Malignant Pleural Mesothelioma from controls. Lung cancer (Amsterdam, Netherlands). 2012; 75(3):326-331.
62. Altomare DF, Di Lena M, Porcelli F, Trizio L, Travaglio E, Tutino M, Dragonieri S, Memeo V and de Gennaro G. Exhaled volatile organic compounds identify patients with colorectal cancer. The British journal of surgery. 2013; 100(1):144-150.
63. Amal H, Leja M, Broza YY, Tisch U, Funka K, Liepniece-Karele I, Skapars R, Xu ZQ, Liu H and Haick H. Geographical variation in the exhaled volatile organic compounds. Journal of breath research. 2013; 7(4):047102.
64. Xu ZQ, Broza YY, Ionsecu R, Tisch U, Ding L, Liu H, Song Q, Pan YY, Xiong FX, Gu KS, Sun GP, Chen ZD, Leja M and Haick H. A nanomaterial-based breath test for distinguishing gastric cancer from benign gastric conditions. British journal of cancer. 2013; 108(4):941-950.
65. Garcia RA, Morales V, Martin S, Vilches E and Toledano A. Volatile Organic Compounds Analysis in Breath Air in Healthy Volunteers and Patients Suffering Epidermoid Laryngeal Carcinomas. Chromatographia. 2014; 77(5-6):501-509.
66. Gruber M, Tisch U, Jeries R, Amal H, Hakim M, Ronen O, Marshak T, Zimmerman D, Israel O, Amiga E, Doweck I and Haick H. Analysis of exhaled breath for diagnosing head and neck squamous cell carcinoma: a feasibility study. British journal of cancer. 2014; 111(4):790-798.
67. Leunis N, Boumans ML, Kremer B, Din S, Stobberingh E, Kessels AG and Kross KW. Application of an electronic nose in the diagnosis of head and neck cancer. The Laryngoscope. 2014; 124(6):1377-1381.
68. Shehada N, Bronstrup G, Funka K, Christiansen S, Leja M and Haick H. Ultrasensitive Silicon Nanowire for Real-World Gas Sensing: Noninvasive Diagnosis of Cancer from Breath Volatolome. Nano letters. 2015; 15(2):1288-1295.
69. Wang C, Ke C, Wang X, Chi C, Guo L, Luo S, Guo Z, Xu G, Zhang F and Li E. Noninvasive detection of colorectal cancer by analysis of exhaled breath. Analytical and bioanalytical chemistry. 2014; 406(19):4757-4763.
70. Amal H, Shi DY, Ionescu R, Zhang W, Hua QL, Pan YY, Tao L, Liu H and Haick H. Assessment of ovarian cancer conditions from exhaled breath. International journal of cancer Journal international du cancer. 2015; 136(6):E614-622.
71. Amal H, Leja M, Funka K, Skapars R, Sivins A, Ancans G, Liepniece-Karele I, Kikuste I, Lasina I and Haick H. Detection of precancerous gastric lesions and gastric cancer through exhaled breath. Gut. 2015.
72. Guo L, Wang C, Chi C, Wang X, Liu S, Zhao W, Ke C, Xu G and Li E. Exhaled breath volatile biomarker analysis for thyroid cancer. Translational research : the journal of laboratory and clinical medicine. 2015.
73. Kumar S, Huang J, Abbassi-Ghadi N, Mackenzie HA, Veselkov KA, Hoare JM, Lovat LB, Spanel P, Smith D and Hanna GB. Mass Spectrometric Analysis of Exhaled Breath for the Identification of Volatile Organic Compound Biomarkers in Esophageal and Gastric Adenocarcinoma. Annals of surgery. 2015.

Table S3. Volatile organic compounds used for model building to discriminate cancer cases and controls or/and showing statistically significant concentrations in exhaled breath between cases and controls in at least 3 independent studies.

Cancer site	LC	LC	LC	LC	LC	LC	LC	LC	LC	LC	LC	LC	LC	LC	LC	LC	LC	LC	LC	LC	LC	LC	LC	LC	LC	LC	LC	BC	BC	BC	BC	BC	BC	BC	HNC	HNC	HNC	CRC	CRC	GC	GC	GC	GC	LVC	MM	OC	PC	TC	Total[a]
Reference number → Volatile compound ↓	[1, 2]	[3]	[4]	[5]	[6]	[7]	[8]	[9]	[10]	[11]	[12]	[13]	[14]	[15]	[16]	[17]	[18]	[19]	[20]	[21]	[22]	[23]	[24]	[25]	[26]	[27]	[28]	[29]	[30]	[31]	[32]	[19]	[33]	[34]	[35]	[36]	[37]	[38]	[39]	[40]	[41]	[42]	[43]	[44]	[45]	[46]	[19]	[47]	
ethenylbenzene														x			x		x				x		x																x			x	x	x			9
heptanal					x						x			x					x				x	x				x					x									x							9
nonanal				x	x						x													x				x										x			x	x				x			9
propan-2-ol		x	x			x		x														x			x		x						x																8
hexanal			x	x	x									x					x					x				x														x							8
butan-2-one	x	x											x		x	x																			x											x			7
2-methylbuta-1,3-diene							x						x	x					x				x											x						x									7
propan-1-ol		x				x	x						x		x	x					x																												7
dodecane			x		x													x																x					x						x				6
pentane							x									x				x			x			x				x																			6
undecane													x	x					x															x		x									x				6
decane														x					x				x																					x	x				5
octanal			x	x																				x				x														x							5
pentanal				x							x		x											x																		x							5
3-hydroxybutan-2-one	x								x				x																															x					4
toluene																	x	x					x																						x		x		4
2,4-dimethylheptane								x									x		x																										x				4
2-methylpentane			x													x							x															x											4
4-methyloctane																	x			x						x												x											4
benzene														x					x				x			x																							4
cyclohexanone					x																										x								x									x	4
decanal			x																																			x				x				x			4
ethanol			x														x																		x	x													4
ethylbenzene		x			x																		x		x																								4
2,2-dimethyldecane																																					x		x								x	x	4
(methylsulfanyl)methane						x		x																		x																							3
1,2,4-trimethylbenzene														x					x				x																										3
1,4-xylene																			x																			x									x		3
1-phenylethan-1-one													x																				x												x				3
2,6,11-trimethyldodecane										x		x																						x															3
VOC-1										x											x													x															3
2-methylbutane													x													x												x											3
3-methylhexane			x																	x									x																				3
3-methylpentane																x										x												x											3
5-(2-methylpropyl)nonane										x		x																				x																	3
6-methylhept-5-en-2-one			x																																					x	x								3
benzaldehyde													x			x																													x				3
butanal		x																						x																		x							3
cyclohexane																			x																			x							x				3
methanol			x				x						x																																				3
methylcyclopentane														x					x																			x											3
prop-2-enenitrile																																				x				x			x						3
propan-2-one							x						x		x																																		3
propanal			x			x																		x																									3
tridecane										x		x																						x															3

VOC-1 = 2,6-di-tert-butylcyclohexa-2,5-diene-1,4-dione. LC-Lung cancer; BC-Breast cancer; HNC-Head and Neck cancer; CRC-Colorectal cancer; GC-Gastric cancer; LVC-Liver cancer; MM- Malignant Mesothelioma; OC-Ovarian cancer; PC-Prostate cancer; TC-Thyroid cancer.

[a]Total number of times when the compound was used for building a classification model or when the concentration between cases and controls were significantly different. Grey color indicates studies which showed significant difference in concentration of volatile compound in exhaled breath between cases and controls, and while color – studies which used compounds for building a classification model.

References

1. Fu XA, Li M, Knipp RJ, Nantz MH and Bousamra M. Noninvasive detection of lung cancer using exhaled breath. Cancer medicine. 2014; 3(1):174-181.
2. Bousamra M, 2nd, Schumer E, Li M, Knipp RJ, Nantz MH, van Berkel V and Fu XA. Quantitative analysis of exhaled carbonyl compounds distinguishes benign from malignant pulmonary disease. The Journal of thoracic and cardiovascular surgery. 2014; 148(3):1074-1080; discussion 1080-1071.
3. Buszewski B, Ligor T, Jezierski T, Wenda-Piesik A, Walczak M and Rudnicka J. Identification of volatile lung cancer markers by gas chromatography-mass spectrometry: comparison with discrimination by canines. Analytical and bioanalytical chemistry. 2012; 404(1):141-146.
4. Filipiak W, Filipiak A, Sponring A, Schmid T, Zelger B, Ager C, Klodzinska E, Denz H, Pizzini A, Lucciarini P, Jamnig H, Troppmair J and Amann A. Comparative analyses of volatile organic compounds (VOCs) from patients, tumors and transformed cell lines for the validation of lung cancer-derived breath markers. Journal of breath research. 2014; 8(2):027111.
5. Fuchs P, Loeseken C, Schubert JK and Miekisch W. Breath gas aldehydes as biomarkers of lung cancer. International journal of cancer Journal international du cancer. 2010; 126(11):2663-2670.
6. Handa H, Usuba A, Maddula S, Baumbach JI, Mineshita M and Miyazawa T. Exhaled breath analysis for lung cancer detection using ion mobility spectrometry. PloS one. 2014; 9(12):e114555.
7. Kischkel S, Miekisch W, Sawacki A, Straker EM, Trefz P, Amann A and Schubert JK. Breath biomarkers for lung cancer detection and assessment of smoking related effects - confounding variables, influence of normalization and statistical algorithms. Clinica Chimica Acta. 2010; 411(21-22):1637-1644.
8. Ma H, Li X, Chen J, Wang H, Cheng T, Chen K and Xu S. Analysis of human breath samples of lung cancer patients and healthy controls with solid-phase microextraction (SPME) and flow-modulated comprehensive two-dimensional gas chromatography (GC x GC). Analytical Methods. 2014; 6(17):6841-6849.
9. Rudnicka J, Walczak M, Kowalkowski T, Jezierski T and Buszewski B. Determination of volatile organic compounds as potential markers of lung cancer by gas chromatography-mass spectrometry versus trained dogs. Sensors and Actuators B-Chemical. 2014; 202:615-621.
10. Song G, Qin T, Liu H, Xu GB, Pan YY, Xiong FX, Gu KS, Sun GP and Chen ZD. Quantitative breath analysis of volatile organic compounds of lung cancer patients. Lung cancer (Amsterdam, Netherlands). 2010; 67(2):227-231.
11. Wang Y, Hu Y, Wang D, Yu K, Wang L, Zou Y, Zhao C, Zhang X, Wang P and Ying K. The analysis of volatile organic compounds biomarkers for lung cancer in exhaled breath, tissues and cell lines. Cancer biomarkers : section A of Disease markers. 2012; 11(4):129-137.
12. Xu H, Wei Y, Zhu L, Huang J, Li Y, Liu F, Wang S and Liu S. Bifunctional magnetic nanoparticles for analysis of aldehyde metabolites in exhaled breath of lung cancer patients. Journal of Chromatography A. 2014; 1324:29-35.
13. Zou Y, Zhang X, Chen X, Hu Y, Ying K and Wang P. Optimization of volatile markers of lung cancer to exclude interferences of non-malignant disease. Cancer biomarkers : section A of Disease markers. 2014; 14(5):371-379.
14. Bajtarevic A, Ager C, Pienz M, Klieber M, Schwarz K, Ligor M, Ligor T, Filipiak W, Denz H, Fiegl M, Hilbe W, Weiss W, Lukas P, Jamnig H, Hackl M, Haidenberger A, et al. Noninvasive detection of lung cancer by analysis of exhaled breath. BMC cancer. 2009; 9:348.
15. Chen X, Cao MF, Li Y, Hu WJ, Wang P, Ying KJ and Pan HM. A study of an electronic nose for detection of lung cancer based on a virtual SAW gas sensors array and imaging recognition method. Measurement Science & Technology. 2005; 16(8):1535-1546.
16. Gordon SM, Szidon JP, Krotoszynski BK, Gibbons RD and O'Neill HJ. Volatile organic compounds in exhaled air from patients with lung cancer. Clinical chemistry. 1985; 31(8):1278-1282.
17. Ligor M, Ligor T, Bajtarevic A, Ager C, Pienz M, Klieber M, Denz H, Fiegl M, Hilbe W, Weiss W, Lukas P, Jamnig H, Hackl M, Buszewski B, Miekisch W, Schubert J, et al. Determination of volatile organic compounds in exhaled breath of patients with lung cancer using solid phase microextraction and gas chromatography mass spectrometry. Clinical chemistry and laboratory medicine : CCLM / FESCC. 2009; 47(5):550-560.
18. Peng G, Tisch U, Adams O, Hakim M, Shehada N, Broza YY, Billan S, Abdah-Bortnyak R, Kuten A and Haick H. Diagnosing lung cancer in exhaled breath using gold nanoparticles. Nature nanotechnology. 2009; 4(10):669-673.
19. Peng G, Hakim M, Broza YY, Billan S, Abdah-Bortnyak R, Kuten A, Tisch U and Haick H. Detection of lung, breast, colorectal, and prostate cancers from exhaled breath using a single array of nanosensors. British journal of cancer. 2010; 103(4):542-551.
20. Phillips M, Gleeson K, Hughes JM, Greenberg J, Cataneo RN, Baker L and McVay WP. Volatile organic compounds in breath as markers of lung cancer: a cross-sectional study. Lancet. 1999; 353(9168):1930-1933.
21. Phillips M, Cataneo RN, Cummin AR, Gagliardi AJ, Gleeson K, Greenberg J, Maxfield RA and Rom WN. Detection of lung cancer with volatile markers in the breath. Chest. 2003; 123(6):2115-2123.
22. Phillips M, Altorki N, Austin JH, Cameron RB, Cataneo RN, Greenberg J, Kloss R, Maxfield RA, Munawar MI, Pass HI, Rashid A, Rom WN and Schmitt P. Prediction of lung cancer using volatile biomarkers in breath. Cancer biomarkers : section A of Disease markers. 2007; 3(2):95-109.
23. Phillips M, Altorki N, Austin JH, Cameron RB, Cataneo RN, Kloss R, Maxfield RA, Munawar MI, Pass HI, Rashid A, Rom WN, Schmitt P and Wai J. Detection of lung cancer using weighted digital analysis of breath biomarkers. Clinica chimica acta; international journal of clinical chemistry. 2008; 393(2):76-84.
24. Poli D, Carbognani P, Corradi M, Goldoni M, Acampa O, Balbi B, Bianchi L, Rusca M and Mutti A. Exhaled volatile organic compounds in patients with non-small cell lung cancer: cross sectional and nested short-term follow-up study. Respiratory research. 2005; 6:71.
25. Poli D, Goldoni M, Corradi M, Acampa O, Carbognani P, Internullo E, Casalini A and Mutti A. Determination of aldehydes in exhaled breath of patients with lung cancer by means of on-fiber-derivatisation SPME-GC/MS. Journal of chromatography B, Analytical technologies in the biomedical and life sciences. 2010; 878(27):2643-2651.

26. Rudnicka J, Kowalkowski T, Ligor T and Buszewski B. Determination of volatile organic compounds as biomarkers of lung cancer by SPME-GC-TOF/MS and chemometrics. Journal of chromatography B, Analytical technologies in the biomedical and life sciences. 2011; 879(30):3360-3366.
27. Ulanowska A, Kowalkowski T, Trawinska E and Buszewski B. The application of statistical methods using VOCs to identify patients with lung cancer. Journal of breath research. 2011; 5(4):046008.
28. Wehinger A, Schmid A, Mechtcheriakov S, Ledochowski M, Grabmer C, Gastl GA and Amann A. Lung cancer detection by proton transfer reaction mass-spectrometric analysis of human breath gas. International Journal of Mass Spectrometry. 2007; 265(1):49-59.
29. Li J, Peng Y, Liu Y, Li W, Jin Y, Tang Z and Duan Y. Investigation of potential breath biomarkers for the early diagnosis of breast cancer using gas chromatography-mass spectrometry. Clinica chimica acta; international journal of clinical chemistry. 2014; 436:59-67.
30. Mangler M, Freitag C, Lanowska M, Staeck O, Schneider A and Speiser D. Volatile organic compounds (VOCs) in exhaled breath of patients with breast cancer in a clinical setting. Ginekologia polska. 2012; 83(10):730-736.
31. Hietanen E, Bartsch H, Bereziat JC, Camus AM, McClinton S, Eremin O, Davidson L and Boyle P. Diet and oxidative stress in breast, colon and prostate cancer patients: a case-control study. European journal of clinical nutrition. 1994; 48(8):575-586.
32. Wang C, Sun B, Guo L, Wang X, Ke C, Liu S, Zhao W, Luo S, Guo Z, Zhang Y, Xu G and Li E. Volatile organic metabolites identify patients with breast cancer, cyclomastopathy, and mammary gland fibroma. Scientific reports. 2014; 4:5383.
33. Phillips M, Cataneo RN, Ditkoff BA, Fisher P, Greenberg J, Gunawardena R, Kwon CS, Tietje O and Wong C. Prediction of breast cancer using volatile biomarkers in the breath. Breast cancer research and treatment. 2006; 99(1):19-21.
34. Phillips M, Cataneo RN, Saunders C, Hope P, Schmitt P and Wai J. Volatile biomarkers in the breath of women with breast cancer. Journal of breath research. 2010; 4(2):026003.
35. Garcia RA, Morales V, Martin S, Vilches E and Toledano A. Volatile Organic Compounds Analysis in Breath Air in Healthy Volunteers and Patients Suffering Epidermoid Laryngeal Carcinomas. Chromatographia. 2014; 77(5-6):501-509.
36. Gruber M, Tisch U, Jeries R, Amal H, Hakim M, Ronen O, Marshak T, Zimmerman D, Israel O, Amiga E, Doweck I and Haick H. Analysis of exhaled breath for diagnosing head and neck squamous cell carcinoma: a feasibility study. British journal of cancer. 2014; 111(4):790-798.
37. Hakim M, Billan S, Tisch U, Peng G, Dvrokind I, Marom O, Abdah-Bortnyak R, Kuten A and Haick H. Diagnosis of head-and-neck cancer from exhaled breath. British journal of cancer. 2011; 104(10):1649-1655.
38. Altomare DF, Di Lena M, Porcelli F, Trizio L, Travaglio E, Tutino M, Dragonieri S, Memeo V and de Gennaro G. Exhaled volatile organic compounds identify patients with colorectal cancer. The British journal of surgery. 2013; 100(1):144-150.
39. Wang C, Ke C, Wang X, Chi C, Guo L, Luo S, Guo Z, Xu G, Zhang F and Li E. Noninvasive detection of colorectal cancer by analysis of exhaled breath. Analytical and bioanalytical chemistry. 2014; 406(19):4757-4763.
40. Xu ZQ, Broza YY, Ionsecu R, Tisch U, Ding L, Liu H, Song Q, Pan YY, Xiong FX, Gu KS, Sun GP, Chen ZD, Leja M and Haick H. A nanomaterial-based breath test for distinguishing gastric cancer from benign gastric conditions. British journal of cancer. 2013; 108(4):941-950.
41. Amal H, Leja M, Broza YY, Tisch U, Funka K, Liepniece-Karele I, Skapars R, Xu ZQ, Liu H and Haick H. Geographical variation in the exhaled volatile organic compounds. Journal of breath research. 2013; 7(4):047102.
42. Kumar S, Huang J, Abbassi-Ghadi N, Mackenzie HA, Veselkov KA, Hoare JM, Lovat LB, Spanel P, Smith D and Hanna GB. Mass Spectrometric Analysis of Exhaled Breath for the Identification of Volatile Organic Compound Biomarkers in Esophageal and Gastric Adenocarcinoma. Annals of surgery. 2015.
43. Amal H, Leja M, Funka K, Skapars R, Sivins A, Ancans G, Liepniece-Karele I, Kikuste I, Lasina I and Haick H. Detection of precancerous gastric lesions and gastric cancer through exhaled breath. Gut. 2015.
44. Qin T, Liu H, Song Q, Song G, Wang HZ, Pan YY, Xiong FX, Gu KS, Sun GP and Chen ZD. The screening of volatile markers for hepatocellular carcinoma. Cancer epidemiology, biomarkers & prevention : a publication of the American Association for Cancer Research, cosponsored by the American Society of Preventive Oncology. 2010; 19(9):2247-2253.
45. de Gennaro G, Dragonieri S, Longobardi F, Musti M, Stallone G, Trizio L and Tutino M. Chemical characterization of exhaled breath to differentiate between patients with malignant plueral mesothelioma from subjects with similar professional asbestos exposure. Analytical and bioanalytical chemistry. 2010; 398(7-8):3043-3050.
46. Amal H, Shi DY, Ionescu R, Zhang W, Hua QL, Pan YY, Tao L, Liu H and Haick H. Assessment of ovarian cancer conditions from exhaled breath. International journal of cancer Journal international du cancer. 2015; 136(6):E614-622.
47. Guo L, Wang C, Chi C, Wang X, Liu S, Zhao W, Ke C, Xu G and Li E. Exhaled breath volatile biomarker analysis for thyroid cancer. Translational research : the journal of laboratory and clinical medicine. 2015.
